# Double‐Orthogonal Gradient‐Based High‐Throughput Screening Platform for Studying Cell Response Toward Combined Physicochemical Biomaterial Properties

**DOI:** 10.1002/smsc.202300172

**Published:** 2023-11-27

**Authors:** Torben A.B. van der Boon, Lisa E. Tromp, Lu Ge, Liangliang Yang, Carlos F. Guimaraes, Philipp T. Kühn, Qihui Zhou, Ruud A. Bank, Theo G. van Kooten, Patrick van Rijn

**Affiliations:** ^1^ Department of Biomedical Engineering FB-40 W.J. Kolff Institute for Biomedical Engineering and Materials Science University of Groningen, University Medical Center Groningen A. Deusinglaan 1 9713 AV Groningen The Netherlands; ^2^ School of Pharmaceutical Science Wenzhou Medical University Wenzhou Zhejiang 325000 China; ^3^ 3B's Research Group - Biomaterials, Biodegradables and Biomimetics, Headquarters of the European Institute of Excellence on Tissue Engineering and Regenerative Medicine University of Minho, AvePark, Parque de Ciência e Tecnologia 4805-017 Barco, Guimarães Portugal; ^4^ ICVS/3Bs Associate Laboratory AvePark, Zona Industrial da Gandra S. Claudio do Barco, Caldas das Taipas 4806-909 Guimarães Portugal; ^5^ School of Rehabilitation Sciences and Engineering University of Health and Rehabilitation Sciences Qingdao 266071 China

**Keywords:** biointerfaces, combinatorial gradients, high-throughput screenings, materiobiology, stem cells

## Abstract

Implant‐associated complications arise due to non‐optimized cell–biomaterial interactions. It is well known that cells respond to their physicochemical microenvironment on 2D interfaces and 3D networks. Attempts to manipulate this interaction target surface parameters such as wettability (W), stiffness (S), and topography (T) to influence cell differentiation, adhesion, and morphology, due to induction of gene activation and protein expression. Investigating the combinatorial influence of all three mentioned parameters simultaneously remains challenging, though most realistic, since all three parameters are inherently present on a surface. Herein, a novel high‐throughput screening technology, which allows investigating the cell response of human bone‐marrow‐derived mesenchymal stem cells toward three varying biomaterial surface parameters simultaneously, is presented. The platform provides efficient screening and cell response readout to a vast amount of combined biomaterial surface properties, in a single‐cell experiment. Surface gradients of aligned wrinkle T, S, and W are orthogonally combined giving four combinatorial surfaces. The screening outcome is validated by translating interesting regions to homogeneous surfaces. Cells are found to behave similar to the screening in terms of adhesion, spreading, and vimentin expression. The technology tremendously supports the identification of optimal surface parameter combinations and potentially addressing many of the current implant‐associated complications.

## Graphical abstract

Tuning cell–biomaterial interfaces provides the opportunity to enhance medical implants and cell‐based technologies. By providing a novel high‐throughput technology, the optimal combined physicochemical properties, stiffness–wettability–topography, are able to be determined to direct cellular behavior. Translations of the physicochemical properties toward macroscale substrates showing the optimum response may ultimately lead to enhanced medical implant design.

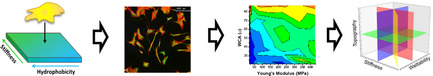

## 1 Introduction

The field of materiobiology and biomaterials discovery is currently devoted to directing cell behavior to stimulate and direct healing processes in native tissue and thereby overcoming adverse effects arising after medical device implantation.^[^ ^1^, ^2^, ^3^ ^]^ Implant‐associated complications, such as impaired tissue integration,^[^ ^4^ ^]^ infections,^[^ ^5^ ^]^ necrosis,^[^ ^6^ ^]^ and fibrosis,^[^ ^7^, ^8^ ^]^ all frequently lead to implant rejection. At the core lies the cell–biomaterial interface, where adhering cells respond to their microenvironment in terms of chemical composition, mechanical properties, structural features on 2D interfaces, but also within 3D gels–or extracellular matrix (ECM)‐like networks.^[^ ^9^, ^10^ ^]^ Complex cellular processes, such as topotaxis,^[^ ^11^ ^]^ chemotaxis,^[^ ^12^, ^13^ ^]^ and mechanosensing,^[^ ^14^, ^15^ ^]^ allow cells to “feel” their microenvironment.^[^ ^16^, ^17^ ^]^ These phenomena activate signaling pathways, which dictate how cells organize their internal structural features, such as the cytoskeleton and focal adhesion complexes. These processes lie at the core of how cells respond to their surroundings, hence their interaction with biomaterials. In attempts to manipulate this interaction, the most commonly assessed biomaterial parameters are surface W,^[^ ^18^, ^19^, ^20^, ^21^ ^]^ S,^[^ ^10^, ^22^, ^23^, ^24^ ^]^ and T.^[^ ^25^, ^26^, ^27^, ^28^, ^29^ ^]^ These parameters have been shown to influence cell differentiation, adhesion, and morphology, driven by induction of specific gene activation and, hence, protein expression.^[^ ^22^, ^30^, ^31^, ^32^, ^33^ ^]^ To this end, various biointerfaces have been designed, including the following: 1) surface structuring:^[^ ^25^ ^]^ in the form of nanopillars,^[^ ^26^ ^]^ microgrooves,^[^ ^27^ ^]^ roughness,^[^ ^33^, ^34^, ^35^ ^]^ predefined combinations of features,^[^ ^36^, ^37^ ^]^ and aligned fibers;^[^ ^28^ ^]^ 2) W: where surfaces are chemically altered to change their water affinity^[^ ^18^, ^19^, ^20^ ^]^ and thereby modulating initial protein adsorption;^[^ ^20^, ^21^, ^38^, ^39^ ^]^ 3) S: in which the internal cross‐linking density of polymer networks cause changes in mechanical properties (Young's modulus),^[^ ^24^, ^40^ ^]^ or hydrogel networks mimicking the ECM.^[^ ^41^, ^42^ ^]^ Control over W, S, and T features enables the manipulation of cellular behavior and thereby enables developing bio‐instructive biomaterial surfaces. While every parameter is important, cells do not merely respond to a single environmental cue, but also to other properties that are inherently present on the surface of a biomaterial. Cells are always subjected to a multitude of these cues simultaneously and to elucidate cell response toward combined influences of biomaterial properties, we need to redefine current methodology as trial‐and‐error approaches are not adequate. To gain insights into more complex cell–material interactions, state‐of‐the‐art techniques in cell studies have been developed, that move in the direction of high‐throughput screening (HTS), a noteworthy overview which was recently presented.^[^ ^1^ ^]^ For instance, the CellPainting assay, which enables high‐throughput morphological profiling of cells regarding eight relevant cellular components, serves as a perfect example of increased cell behavioral insight, keeping efforts to a minimum.^[^ ^43^ ^]^ The general aim in the field is to increase complexity and number of cell–material interaction studies without the constraints of increasing experimental and analytical efforts. For these purposes, some pioneers in the field have developed new approaches to biomaterials discovery.^[^ ^44^, ^45^, ^46^, ^47^, ^48^ ^]^ Among these are endeavors such as those of Langer et al. who developed polymer microarrays to simultaneously study over 1,700 human embryonic stem cell–material interactions leading, for instance, to the identification of materials that allow for high levels of differentiation into cytokeratin‐positive cells.^[^ ^44^ ^]^ Following a similar strategy, Alexander et al. developed a polymer microarray for high‐throughput discovery of biomaterials,^[^ ^49^ ^]^ leading for instance to the discovery of new materials resistant to bacterial attachment,^[^ ^48^ ^]^ and to synthetic substrates beneficial for human pluripotent stem cell expansion.^[^ ^50^ ^]^ Focusing on surface T, de Boer et al. aimed at increasing insights into biomaterial–cell interactions through in silico designed topographical libraries for the identification of ideal surface topographies for any application of interest.^[^ ^36^, ^37^ ^]^ Expanding further the potential of such HTS platforms, combinatorial platforms incorporating more surface parameters have been developed in the form of the ChemoTopoChip, utilized for studying immunomodulatory osteoinductive biomaterials, and the ChemoArchiChip which led to discovering that macrophage attachment and phenotype can be tuned via highly specific combinations of size, geometry, and material.^[^ ^51^, ^52^ ^]^ These examples showcase and stress the need to look at combined influences of material chemistry and surface T. A different approach to libraries of distinct surface properties is the use of surface parameter gradients, where a parameter is gradually altered from one end of a spectrum to the other, which allows to investigate cell response to a large span of different cell–biomaterial interfaces, on the same material. Following a similar combinatorial approach and increasing complexity, orthogonal gradients have been used to investigate two parameters simultaneously, such as W/T^[^ ^53^ ^]^ and W/S,^[^ ^54^ ^]^ but also physicochemical–biological property combinations such as orthogonal gradients of peptides mimicking cell–cell or cell–matrix interactions into a 3D hydrogel system, to investigate the influence of these peptides on cartilage formation.^[^ ^55^ ^]^ Such high‐throughput cell‐screening systems are accelerating biomaterials discovery and illustrate the need to go even beyond two‐parameter systems by increasing the combinatorial physicochemical surface parameter combinations.

**Figure 1:**
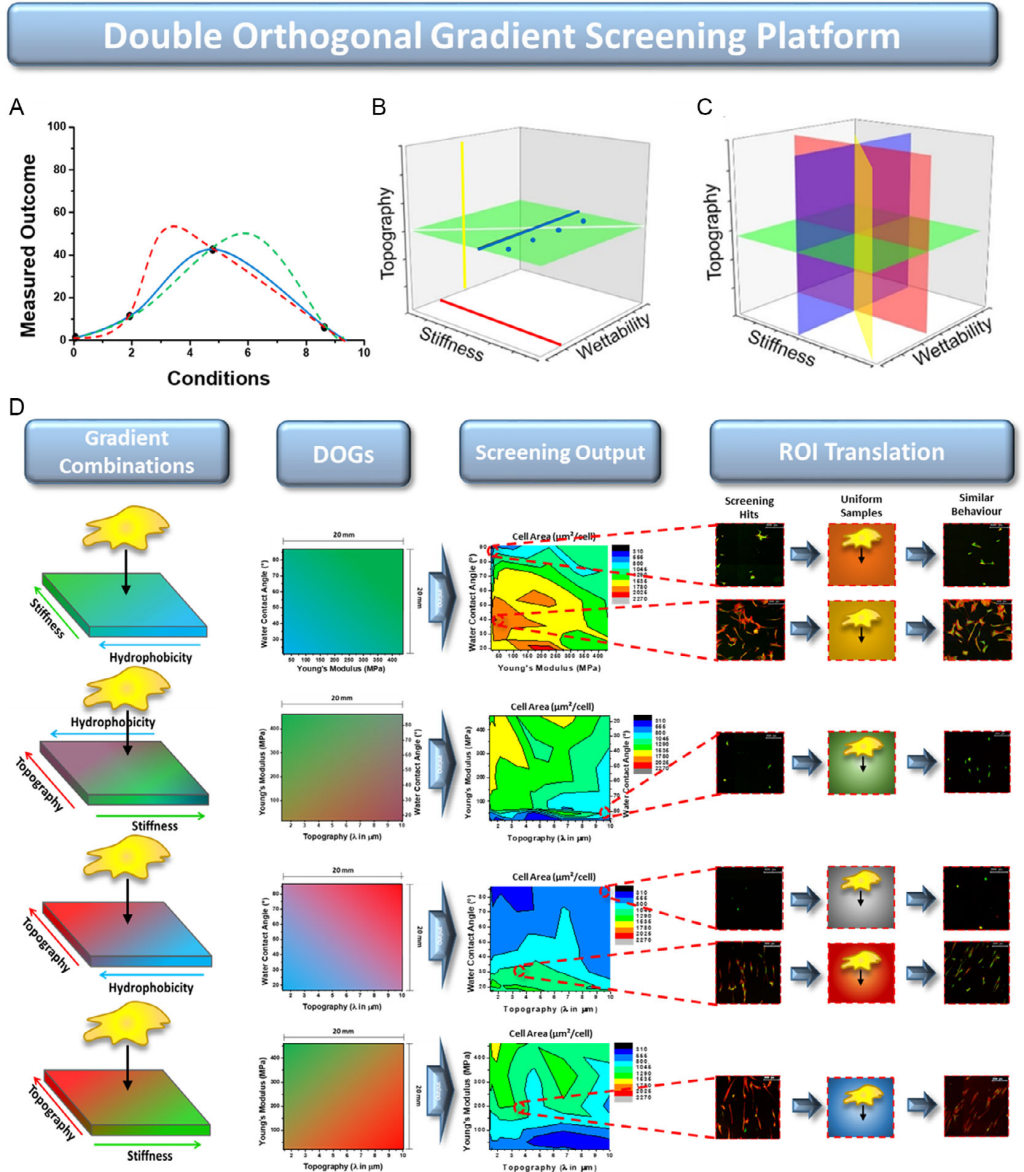
A) Illustrative graph showing that using a gradient instead of a limited sampling approach will reveal a certain optimum, otherwise left undiscovered. Using a standard fit on a limited set of data points (blue line) might give a faulty indication of where an optimum actually could be (red or green lines). B) Representative illustrations of the parameter combinations obtained by a single gradient in wettability (blue line), topography (yellow line), stiffness (red line), and a double‐linear gradient in wettability and stiffness (white line), as well as a double‐orthogonal gradient (DOG) of stiffness and wettability (green plane). C) All other parameter combinations make up the complete platform and accessible data to screen for in single‐cell experiments. D) DOG‐screening platform overview showing, from left to right, the different types of parameter combinations, their respective characterization data, screening output in terms of cell area, and the identification, translation, and verification of screening hits.

Here, we present our combinatorial cell‐screening technology, which facilitates the investigation of cell–biomaterial interactions in the context of not two, but four uncoupled, double‐orthogonal, and physicochemical surface parameter gradients. The technology allows for HTS of cell response toward a vast amount of surface parameter combinations as well as offering high‐translational control of identified beneficial parameter combinations toward optimized biomaterial surfaces, an aspect often difficult to achieve. Our double‐orthogonal gradient (DOG)‐screening technology utilizes surface gradients of either W, S, or T. The huge benefit of using a gradient over a more traditional sampling approach, is that it reveals the real optimum rather than a possible pseudo‐optimum (**Figure** **1**A). In addition to linear W gradients,^[^ ^18^ ^]^ we are able to generate gradients in S^[^ ^54^ ^]^ and T,^[^ ^56^, ^57^ ^]^ as well as combined double‐linear gradient and DOGs of those (Figure 1B), all on the same material. Note that overall, a single dot, a line and a plane (Figure 1B) represent single studies with the same experimental effort, but differing tremendously in complexity and data output of which the plane is the only one providing information on all three parameter combinations simultaneously. In this work, we describe the methodology of combining the three mentioned surface parameter gradients, to create a holistic, high‐throughput cell‐screening platform, which incorporates every parameter combination, within their tunable boundaries (Figure 1C). The general outline of the DOG platform is described in Figure 1D. The platform represents a combination of surface parameter gradients of either 1) aligned nano/microtopography, 2) surface S, and 3) surface W (hydrophobicity). The combinatorial gradients are created on 20 × 20 mm polydimethylsiloxane (PDMS) substrates, using multistep sequential air plasma treatments. After platform characterization, human bone‐marrow‐derived mesenchymal stem cells (hBM‐MSCs) are seeded and cultured onto the DOGs, fixated, visualized by immunostaining, and imaged in an automated fashion, resulting in a fast and detailed screening overview of the cell behavior of interest. The screening overview is visualized by easily interpretable heat maps and additional 3D representations of all screening data combined, per assessed cell behavior. The heat maps and 3D representations aid in visually assessing the cell response to all different regions of the platform and allows for the identification of regions of interest (ROIs). In such ROIs, depending on the biological question at hand, the observed cell behavior can be directly connected to the exact combinatorial surface parameter values present at that specific position on the DOG‐screening platform. Previously, it was already stressed that “for gradient approaches that are applied as rapid screening methods to assess cell response, it is important to validate the results in comparison to uniform sample formats”,^[^ ^58^ ^]^ hence to verify the screening outcome of the current study, a “hit” or ROI is identified and translated to single, homogeneous, substrates bearing the exact same parameter combination. The influence of these parameter combinations on cell behavior is studied again in identical cell culture studies and analysis. This allows for the direct comparison and validation of the observed cell response in the cell screening with their behavior observed in the translation experiments. The translation requires careful reidentification of treatment input parameters. Once identified and verified, these input parameters can directly be used in generating samples and potentially find their way into medical implant surface design. As our system incorporates four DOGs in pursuit to elucidate cell response to three distinct stimuli simultaneously, it provides more insights than conventional single‐ and double‐parameter cell‐screening systems. We envision this technology to tremendously aid the development of better performing, bio‐instructive medical implant surfaces.

## 2 Results

### 2.1 Platform Preparation

**Figure 2:**
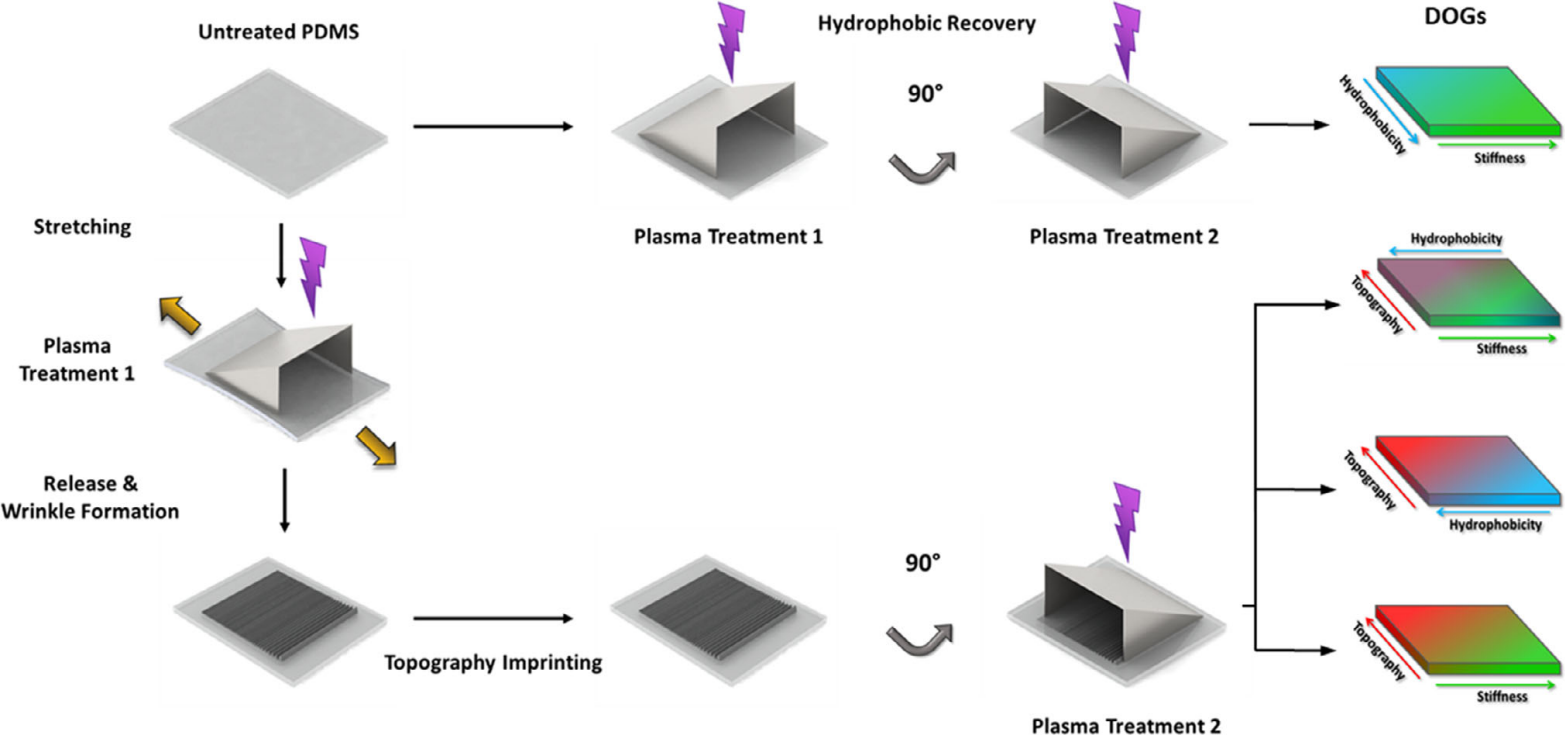
DOG platform generation overview, showing sequential plasma treatments resulting in all possible DOG combinations, starting from an untreated piece of polydimethylsiloxane (PDMS) in the top left‐hand corner.

The three surface parameters: S, W, and W, form the basis of the DOG‐screening platform, which can be combined and tuned using several sequential plasma treatments (**Figure** **2**). The first step comprises a shielded surface plasma oxidation of untreated PDMS to generate the first gradient, resulting in a S gradient (Young's modulus = 17–465 MPa, measured by atomic force microscopy [AFM]) having a coupled gradient in W (87°–19° water contact angle [WCA]) in opposite direction (S/W) (Figure S1A, left, Supporting Information). For the W gradient to be oriented orthogonally to this S gradient, the surface requires additional modification to make the surface hydrophobic again. An overnight silanization reaction with trichloro(propyl)silane (98%) in a desiccator under vacuum ensures that the hydrophilic surface is transformed back into a hydrophobic one (Figure S1B, Supporting Information). Subsequently, by placing the shielding mask perpendicular to the S gradient, and by using a second set of milder plasma conditions, a single W gradient in the same range (18°–91° WCA) is generated, without altering the surface S of the sample (Figure S1A, right, Supporting Information), resulting in the S–W DOG (Figure 2). All other DOGs include a gradient in aligned, wrinkled surface W (*λ* = 1.23–10.10 μm; *A* = 197–2436 nm) (Figure S1C, Supporting Information) in one direction, and one of the three mentioned gradients (S, W, S/W) in the perpendicular direction. The W gradient, the wrinkle gradient, is generated by stretching the PDMS with subsequent shielded plasma treatment and release of the strain. However, to be able to generate the S, W, and S/W gradients on the W gradient, an imprint of the W gradient is made via imprinting lithography by pouring liquid PDMS on top and subsequently letting it cure.

After removal of the template, the W gradient is available in pristine PDMS and the other gradients can be applied in the same fashion as described before generating the DOG of: S‐W, T–S, T–W, T–S/W (Figure 1D and 2). The conditions for the W gradient on the T–W combination slightly differ from the S–W combination and are described in detail in Experimental Section. All four DOG combinations were generated on 20 × 20 mm surface areas. The exact methodology and all data on surface characteristics can be found in Experimental Section and Supporting Information.

### 2.2 Screening and Analysis

**Figure 3:**
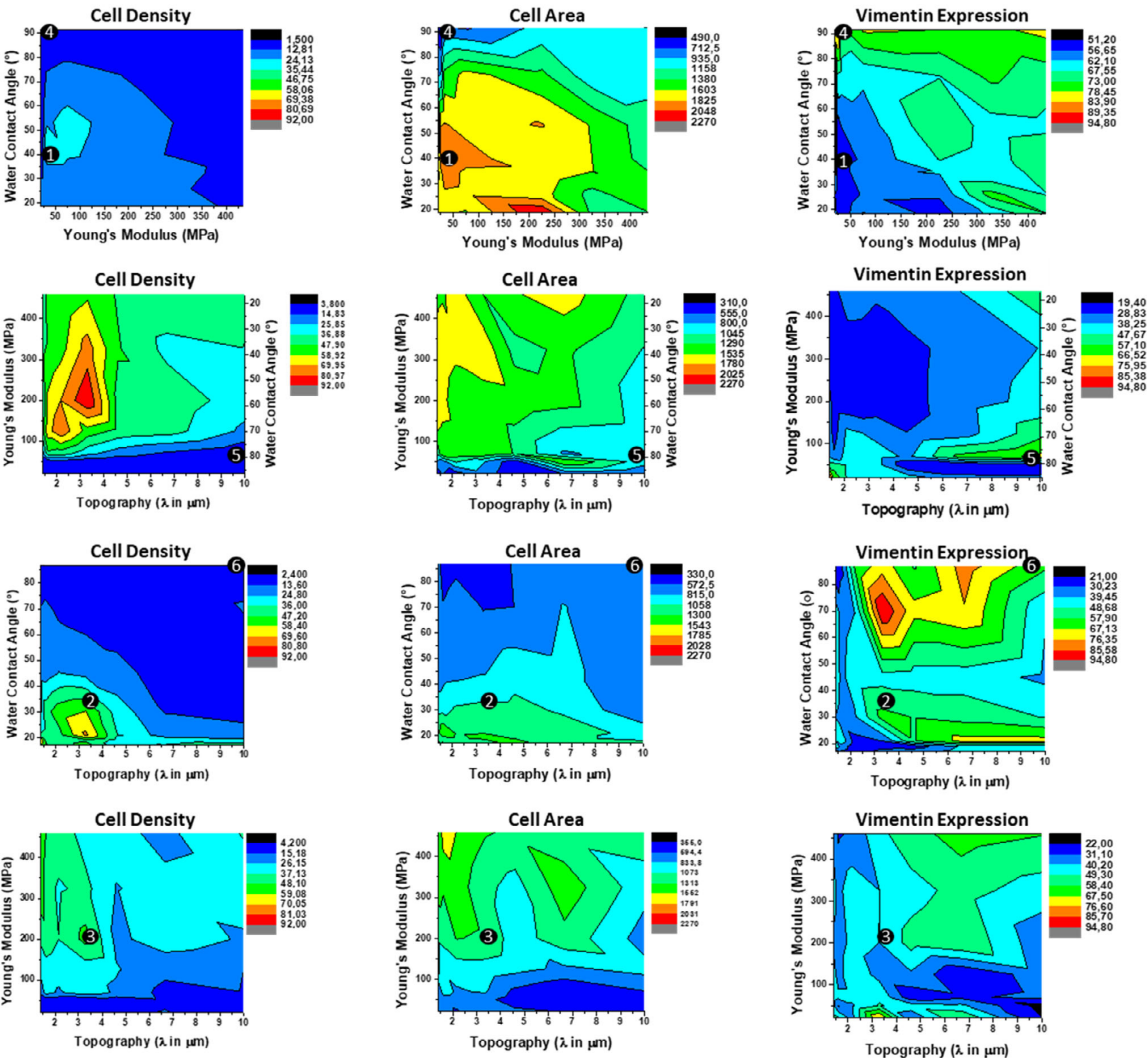
Cell screening overview showing heat maps (*n* = 3) of where cells attached (cell density [cells mm^−^^2^]), their spreading (cell area [μm^2^]), and vimentin expression (A.U.). Six regions of interest (ROIs) are indicated by the numbered black dots, which represent higher and lower values of cell numbers, size, and protein expression. These ROIs were later translated to cell studies on homogeneous samples.

The complete DOG‐screening platform samples were prepared for initial 24 h adhesion experiments with hBM‐MSCs. Cells were seeded and cultured for 24 h and cells were imaged using a TissueFAXS automated fluorescence microscope that can automatically image every area on the surface and join these together into one image in which one can zoom in until the single‐cell level. A selected grid of 7 × 7 regions of analysis (4 mm^2^ per region) was used to analyze the complete 20 × 20 mm^2^ DOG surfaces, taking into account the exponential trend of the gradients. This proof‐of‐concept cell‐screening investigated MSC behavior, concerning cell adhesion, cell spreading, and vimentin protein expression. MSC nucleus visualization with 4′,6‐diamidino‐2‐phenylindole (DAPI) staining allowed to assess cell coverage on the surface, and TRITC–phalloidin cytoskeleton staining enabled cell spreading and surface area assessment. Vimentin protein expression was included in this proof‐of‐concept cell‐adhesion screening, as this intermediate filament is profoundly associated with cell adhesive and migratory behavior and cytoskeletal organization.^[^ ^59^, ^60^, ^61^ ^]^ The fluorescent signal was normalized to the number of cells analyzed and translated into 2D heat maps (**Figure** **3**; individual screening heat maps found in Figure S3–S5, Supporting Information). Visual assessment of the screening data enables to identify ROIs, where specific combinations of the surface parameters dictate certain cell behavior. TissueQuest software was used for quantitative analysis of the fluorescent signal and Origin9 software allowed for generating the heat maps, in which we identified ROIs that displayed opposite behavior. As observed in Figure 3, a higher cell density is observed in regions 1–3 (38, 46, and 49 cells mm^−^^2^, respectively) as compared to regions 4–6 (4, 10, and 6 cells mm^−^^2^, respectively). Higher cell areas and lower expression of vimentin coincided with higher cell density while regions of lower cell densities showed smaller cell sizes and higher expression of vimentin. In regions 1–3, cells ranged in size from approximately 1200 to 2000 μm^2^, while cells in regions 4–6 ranged from approximately 400 to 800 μm^2^.

**Figure 4:**
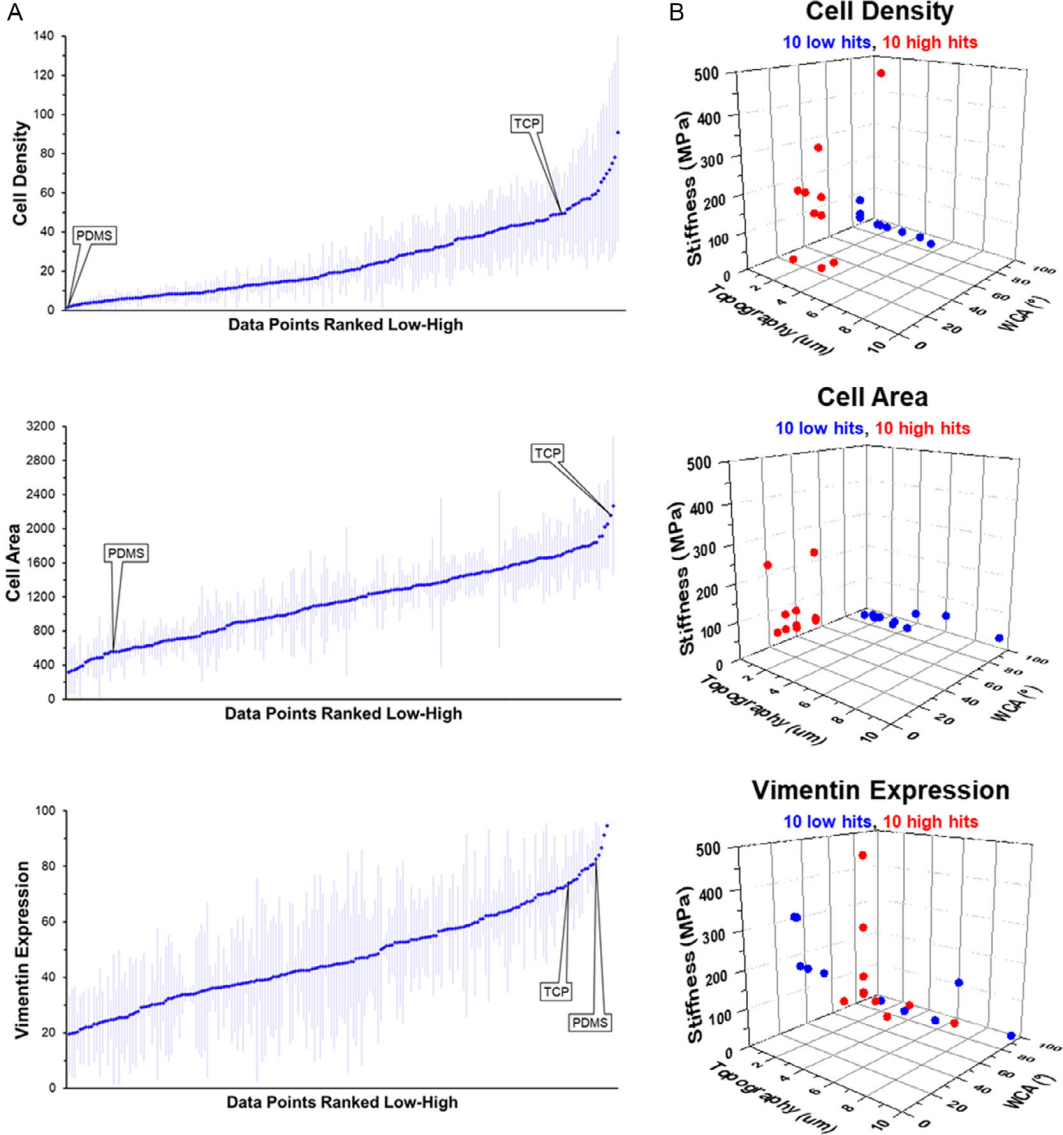
A) Comprehensive representations of all screening data ranked from low to high per assessed cell behavior (*n* = 3). B) 3D representations of the 10 highest and lowest hits per category to visually identify optimal surface parameter combinations (average values, *n* = 3).

A ranking from low‐to‐high for all 588 conditions per three assessed cell behaviors on the DOG platform is depicted in **Figure** **4**A, shown per assessed cell behavior, and included are controls on PDMS and tissue culture polystyrene. The 3D representations of the 10 highest and 10 lowest values for all possible parameter combinations are shown in Figure 4B (3D visualization of all ranked data is shown in Figure S6, Supporting Information). These representations allow to visualize which combinations of surface parameters evoke the specific cell response. As can be observed, the lowest cell densities were found at different surface topographies, but mostly at the same W and S values. Higher cell densities were found at lower WCAs and smaller topographies, but spread out over different S values.

When assessing cell area, the smallest area per cell was found on the higher WCAs and spread out over different topographies, predominantly at lower surface S. The largest cell areas were found in a region between 20° and 60° WCA, mostly on the softer side of the surfaces, at flat topographies. Looking at expression of vimentin, the highest expression was found at the most hydrophobic parts of the screening platform, spread out over different topographies and different S's. Lower expression of vimentin was found spread out over all parts of the surfaces, at different WCAs, different topographies, and different S's.

**Figure 5:**
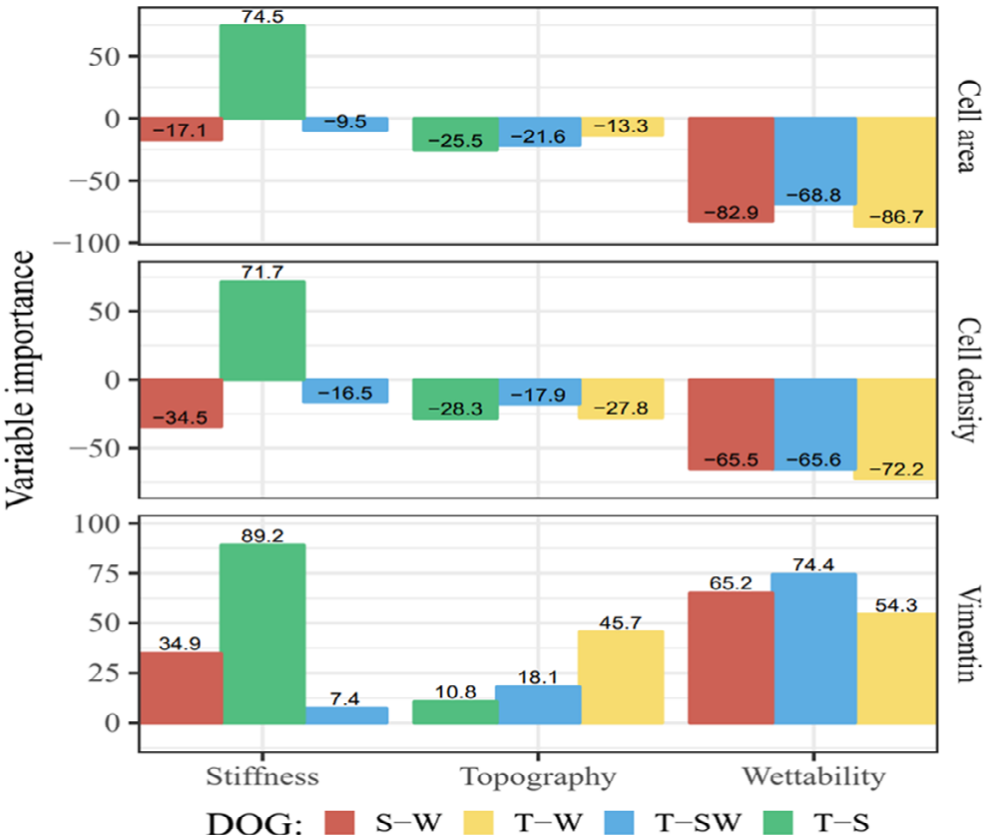
Results of partial least square analysis. Plotted are the relative importance (in %, y‐axis) of each of the surface parameters stiffness (in MPa), topography (in μm), and wettability (water contact angle [WCA] in °) (x‐axis) on the assessed cell behavior (cell density [cells mm^−^^2^]), cell area [μm^2^], and vimentin expression [A.U.]), for each of the DOGs. Positive values represent a positive correlation and negative values represent a negative correlation.

To understand more clearly the relationship between the surface parameters and the observed cell behavior and to assess which (combinations) of the surface parameters had a more dominant effect, partial least squares (PLSs) analysis was performed (**Figure** **5**). Scatter plots that visualize the trends in the dataset are shown in Figure S7, Supporting Information). This figure shows the contributions of each variable relative to the other variables, per DOG (data on R^2^ values and number of components used are shown in Figure S8, Supporting Information). Assessing the influence of surface S, it seems that throughout the three assessed cell responses, it had the most predominant effect in combination with surface T. In combination with W, however, the relative importance of S was less, as was the case on surfaces where all three surface parameters were combined. Surface S seemed to have a mostly positive correlation, with an increase in surface S, cell density, cell area, and vimentin expression also seemed to increase. Looking at surface T, the relative importance seems little when compared to the other two surface parameters. Only in terms of vimentin expression, T in combination with W seemed to have a larger, positive, correlation. A decrease in surface T seemed to decrease cell density and cell area, which was correlated with an increase in vimentin expression. Assessing the relative importance of surface W, it is clearly observable that it had a predominant influence on all types of cell behavior, on all the DOGs in which it was included, regardless of the combination with the other surface parameters. W, in which a higher value for WCA indicates a more hydrophobic surface, seemed to have a negative correlation with cell density and cell area, which could again be correlated to an increase in expression of vimentin.

### 2.3 Translation and Validation

**Figure 6:**
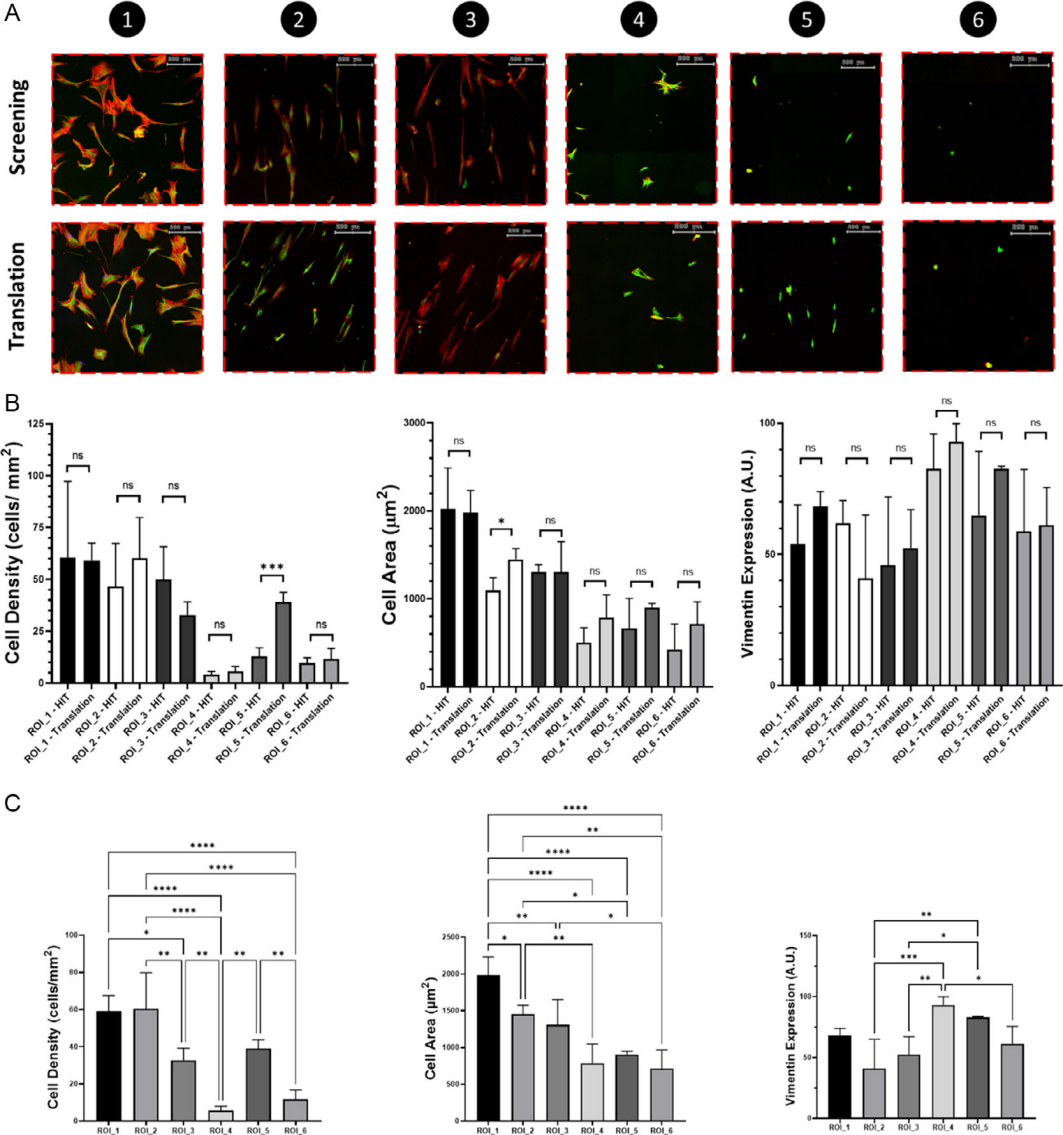
A) Fluorescent images of cells in screening and translation experiments, showing nucleus (blue), cytoskeleton (red), and vimentin protein (green), scale bar = 200 μm. B) Comparative bar plots of screening and translation experiments showing from left to right: cell density, cell area, and vimentin expression. The white bars represent a “hit” identified from the screening data, and the grey bars represent data obtained from the homogeneous, translation experiments. C) Statistical analysis of translation experiments (*n* = 4), showing all translated regions (1–6).

To validate the screening outcome, the material properties associated with the selected ROIs in Figure 3 were translated to homogenous substrates. Subsequently, cell culture studies identical to those conducted in the screening were performed on these substrates. Data of the homogeneous translation experiments was collected in the same manner as in the screening experiment. Fluorescent visualization of complete DOG screening samples, including magnified comparison of ROIs and translated data can be found in Figure S9–S12, Supporting Information. As can be observed from the comparative data for screening and translation in **Figure** **6**B, cell adhesion behavior, in terms of number of cells adhering, was identical for almost all regions. Translated regions 1–3 ranged from approximately 35 to 50 cells mm^−^^2^ (no statistically significant difference between screening and translation), and 4 and 6 ranged from approximately 5–10 cells mm^−^^2^, with deviating value of 40 cells mm^−2^ in ROI_5. The same trend can be observed for the cell area, where cells were spread out and relatively larger in those regions that showed more adhering cells (1–3: 1200–2000 μm^2^), as compared to the regions of lower cell density (4–6: 700–1000 μm^2^), though differing slightly in ROI_2. In terms of vimentin expression, most hits and translation regions corresponded well, although accompanied by a slightly larger deviation. Regions 1–3 corresponded with a high expression (no statistically significant difference between screening and translation), and regions 4–6 with a low expression (no statistically significant difference between screening and translation). Results of statistical analysis performed on the data originating from the translated, homogeneous experiments are observed in Figure 6C. All selected and translated ROIs were compared with each other in terms of provoked cell behavior. All data showed significant differences in observed cell behavior between the translated specific surface parameters, except for vimentin expression in ROI_1.

## 3 Discussion

With the high‐throughput technology presented here, we provide a clear method to elucidate the effects of combined biomaterial properties on cellular behavior, which allows for tailoring the material–cell interaction. The platform connects combinations of surface gradients in an orthogonal fashion leading to DOGs of S–W, W–W, W–S, and W–S/W. Each DOG holds a vast amount of unique surface parameter combinations, within the limits of the tunable span of the gradients themselves, which are able to be studied in great detail when required. After the screening of hBM‐MSC adhesion behavior to this vast number of physicochemical surface parameter combinations, we gain tremendous insights into the cell–biomaterial interactions. Results of the PLS analysis aided in identifying the influence of the surface parameters, relative to one another. In this screening study, it seemed that surface W was the most dominant influence on the cell response. The *T*–*S* DOG is the only surface which does not include surface W, and there the influence of surface S was dominant. However, as soon as S was combined with W, the relative influence of S diminished. Moreover, T seemed to have little influence as compared to the other parameters. This was the case in all combinations, except when combined with surface W. When assessing only W relative to the other parameters, it showed high relative importance in all parameter combinations. Taken together, we can conclude that surface W had the most dominant effect on cell response, regardless of the parameter combinations. Moreover, the results of the PLS analysis showcases the importance and strength of a screening platform that includes all three surface parameters, since the relative importance of each parameter changes when combined with another.

The readout of the screening heat maps and translational step of “ROI” to homogeneous surfaces is an essential asset, which allowed us to verify the cell behavior observed in the screening. This revealed hBM‐MSCs to show similar behavior in both the screening and translated studies. Nearly all screening and translation experiment data were an identical match among the selected regions. However, in few aspects, the screening and translation data did not match completely. A discrepancy was found in cell density at ROI_5 compared to the translated substrate bearing the same physicochemical surface parameter combinations, which could possibly be attributed to cell seeding variation, but more likely due to the effect of the gradients themselves. In literature, similar observations of differences between gradient samples and homogeneous samples were attributed to cell–cell communication or differences in protein production from neighboring cells.^[^ ^58^ ^]^ ROI_5 is located on a low‐density spot with areas of higher density in close proximity and those areas also induce higher cell spreading. It is likely that cells migrate toward those areas in response to external cues originating from other cells, or simply by locating a better anchorage spot, leaving a somewhat depleted area. This is not possible on the homogenous substrate as there is no direct incentive for migration leading to a higher cell density. As mentioned before, vimentin is greatly involved in adhesion and cell migratory behavior.^[^ ^59^, ^60^, ^61^ ^]^ The higher vimentin expression in ROI_5 on the homogenous substrates, as compared to the more favorable regions, supports this expression behavior. However, in‐depth studies on cell migration, cell–cell communication and expression of signaling molecules should be performed to confirm this. Moreover, the fact that the screening data at this specific site is less accurate due to lower cell numbers might also have contributed to this discrepancy. However, even with this observed discrepancy in cell density, the cell behavior in terms of cell area and vimentin expression is observed to be similar, revealing that the exact same parameter combinations elicit the same adhesion behavior of MSCs. This means that, regardless of deviations in number of cells, the translated surface parameter combinations evoke similar cell–biomaterial interaction.

In ROI_2, we observed, although relatively small, a mismatch considering cell area. This could again originate from the difference in cells that, on the one hand reside on a multiple gradient surface, and on the other hand on homogeneous substrates. Slight deviations in surface parameters within the selected areas for data analysis in our screening experiments might give rise to discrepancies between a “hit” and the “translation” data. Practically, we aim to select a considerate number of cells for analysis, by determining a standardized surface area. However, this means that within this region, a surface gradient is still present, though minimized. This effect is most prevalent in the steeper regions of the gradients. This phenomenon poses a current limitation in our analysis approach and not in the platform itself, which will be addressed in the continuous improvement of the platform in terms of analysis and expansion toward other materials than silicone.

Evidently, the approach of combining decoupled biomaterial surface parameters, in a gradient fashion, leaps away from conventional trial‐and‐error, non‐combinatorial experimental systems. The technology we have presented here also drastically increases insights to be gained in cell–biomaterial interactions, as compared to other state‐of‐the‐art screening methodologies, as it utilizes not two, but three distinct biomaterial surface parameters. For instance, in our previous work, we found that surface T and material chemistry can generate synergistic effects on the response of hBM‐MSCs, varying for each material chemistry used in the study.^[^ ^57^ ^]^ Furthermore, when investigating two‐parameter contributions of S and W, we observed MSCs to display altered behavior as compared to solely investigating the W of the biointerface with a homogenous S.^[^ ^18^ ^]^ Both showcase the interdependent relationship of multiple surface parameters on cell response. By incorporating three surface parameters in the screening platform presented in this work, we have improved our previous HTS system and are another step closer to unraveling the complex interactions at the cell–biomaterial interface.

However, there are still limitations and optimizations possible, as already briefly highlighted. Logically, the presence of paracrine factors originating from cells on other parts of the gradients in the initial screening experiments might result in some deviations depending on the type of experiment and type of cell used. Additionally, in terms of translational methodologies, there is still room for some practical optimizations. Taking all screening and translation data into consideration, we can state that, although there are some relatively large deviations in cell density in between the individual screening experiments, this still matches with the results on homogeneous substrates, and does not influence the outcome of the other types of cell behavior assessed. Overall, we conclude that our technology of creating a 3‐parameter combinatorial cell‐screening platform is envisioned to tremendously advance and contribute to high‐throughput discovery of optimal biomaterial surfaces. Especially, the exact translation of the findings from the initial screening to homogeneous substrates confirms that this proof‐of‐concept study shows the potential of the platform to discover bio‐instructive capabilities toward developing better medical implants.

## 4 Experimental Section

4.1

4.1.1

##### PDMS Sample Preparation

The silicone rubber substrates were prepared using a commercially available, Sylgard 184 elastomer kit, following the supplier's guidelines (Dow Corning/DowDuPont). The monomer and cross‐linker were mixed in a 10:1 weight ratio, respectively. The mixture was stirred thoroughly for at least 5 min and 18.0 g of the mixture was poured in square (12 × 12 cm), polystyrene petri dishes (Greiner Bio‐One) and left to rest on a workbench at room temperature for 3 h, to let gas bubbles escape the mixture. Using 18.0 g of the mixture in the mentioned petri dishes yields a PDMS film of 1 mm thickness. After degassing, the samples were cured overnight at 70 °C and ambient pressure, to expedite the cross‐linking process. After cross‐linking, the PDMS was cut into 2.5 × 3.0 cm pieces to be mounted into the home‐made stretching device, and to allow ample space for the 2.0 × 2.0 cm DOGs to be generated on top of the samples. To be compatible with cell culture plates, all samples were cut to meet the dimensions of the inner radius of commercially available GRIENER Bio‐One CELLSTAR multiwell 6‐well plates, using a custom‐made circular puncher.

##### Shielded Air Plasma

A Diener Electronic ATTO plasma oven with adjustable air flow was used to generate the charged gas. Charged gas oxidized the surface of PDMS, resulting in an alteration of the surface S^[^ ^62^ ^]^ and chemistry.^[^ ^63^ ^]^ By placing an angular mask on top of the PDMS substrate surface before plasma treatment, a shadow effect was obtained during treatment, resulting in the largest exposure to the plasma on the open side of the mask and gradually decreasing the exposure toward the closed side of the mask. This approach ensured a gradient in exposure to the air plasma. The different types of gradients all required a different parameter setup.

##### Single‐T Gradient

To generate T gradients, we used the same stretch‐and‐release approach as described by us before,^[^ ^56^, ^64^ ^]^ using a 2 × 2.6 cm mask with an angular aperture of 30° and stretching the substrate to 120% of its original length. The stretched samples, having the mask on top, were placed inside the plasma system in the same orientation and at the same position each time a sample was generated. The samples were treated at a pressure of 10–12 mTorr for 650 s. After the samples were taken out, the mask was removed and the stretching force was released gently, upon which wrinkle formation occurred (Figure S1C, Supporting Information). All T samples received a posttreatment at 150 mTorr for 600 s to ensure homogeneous surface charge and left to rest at room temperature for 5 days, before entering the T imprinting step. This last step was crucial to enable samples to be detached from one another after the imprinting procedure.

##### Imprinted Single‐T Gradient

To create a DOG having the T present as one of the gradients, a freshly cured exact copy of the initial plasma‐treated T sample was created. This was a critical step to ensure the T gradient solely comprised a T gradient, without it being altered for its S and water affinity. As described in our previously established method,^[^ ^65^ ^]^ after the 5 days of resting step, the original T gradient sample was firmly attached to the bottom of a Greiner Bio‐One CELLSTAR round petri dish (d = 60 mm; h = 15 mm) with T features facing upward. After this, exactly 5.0 g of freshly mixed liquid PDMS was poured over the T sample, covering the surface completely with a 1 mm thick layer. This “sandwich” was left to rest at room temperature for 3 h, in order for gas bubbles to escape the liquid PMDS, as well as to ensure the mixture reached the bottom of the smallest, nanosized wrinkles. After this resting step, the samples were cured in a heating oven until fully cross‐linked. Next, the T copy, which was the inverse of the original, but since it was a wave pattern, it remained the same, was gently peeled off and a single‐T gradient was left, having the same S and wetting properties, as freshly cured PDMS (data in Figure S2, Supporting Information). This T gradient could now receive the second treatment of choice, to gain one of the desired DOGs.

##### Unidirectional Double Gradient

Another custom‐made 2 × 2 cm mask with an angular aperture of 30° was used for generating all other surface gradients, and was placed on top of the sample in the same fashion as described before, either for generating the first gradient, or for the second gradient. In the latter case, it was placed perpendicular to the first surface gradient, as can be seen in Figure 2. The plasma conditions for generating a double‐linear gradient of S and W were at a pressure of 20 mTorr and a treatment time of 40 s (gradient in Figure S1, Supporting Information).

##### Single‐S Gradient

To generate a single‐S gradient, first the double‐linear gradient of S–W was created as described before. To form the S gradient without any contributions to W, an overnight silanization reaction was performed to ensure a homogeneous hydrophobic surface (Figure S1B, Supporting Information). After the first plasma treatment that forms the double‐linear S–W gradient, samples were directly placed into a desiccator containing a vial with a total amount of 50 μL (0.06 g, 0.34 mmol) trichloro(propyl)silane (98%, Sigma–Aldrich). Vacuum was applied overnight to modify the PDMS surface and yielded a hydrophobic surface again. This left only the S gradient present at the surface, and the sample ready to receive a second treatment.

##### Single‐W Gradient

Generating a single‐W gradient, without altering the surface S, required milder plasma conditions. A pressure of 1000 mTorr and a treatment time of 60 s, using the 20 × 20 mm mask with an angular aperture of 30°, yielded a single‐W gradient, without altering the surface S of the PDMS (Figure S1A, Supporting Information). For the single‐W gradient to be created on the surfaces which underwent overnight silanization, the same treatment conditions were applied, only for a shorter amount of time (30 s).

##### DOG

By combining and sequentially treating PDMS samples with the previously described single‐ or double‐linear gradients under a 90° angle relative to one another, DOGs of T–S, T–W, T–S/W, and S–W were created.

##### DOG: T–S

First, the T gradient was created as described in “*Single Topography Gradient”.* An exact inverse copy of this was created as described in “*Imprinted Single Topography Gradient*”, which received a second treatment perpendicular to the direction of the T gradient as described in “*Unidirectional Double Gradient”*. This was left to rest for 24 h in order for the hydrophobic recovery process to yield a hydrophobic surface again, which was the nature of PDMS. This process was, however, less applicable when subsequently the W gradients needed to be applied for which the silanization reaction should be used.

##### DOG: T–W

First, the T gradient was created as described in “*Single Topography Gradient”.* An exact inverse copy of this was created as described in “*Imprinted Single Topography Gradient*”, which received a second treatment perpendicular to the direction of the T gradient as described in “*Single Wettability Gradient”*. This left only the T and W gradients present at the sample surface. This second treatment was performed closely before cell culture studies and directly after treatment the freshly treated DOG was immersed in milliQ water, to ensure the polar environment inhibited the hydrophobic recovery process.

##### DOG: T–S/W

First, the T gradient was created as described in “*Single Topography Gradient”.* An exact inverse copy of this was created as described in “*Imprinted Single Topography Gradient*”, which received a second treatment perpendicular to the direction of the T gradient as described in “*Unidirectional Double Gradient”*. This second treatment was performed closely before cell culture studies and directly after treatment the freshly treated DOG was immersed in milliQ water, to ensure the polar environment inhibited the hydrophobic recovery process.

##### DOG: S–W

First, the S gradient was created as described in “*Single Stiffness Gradient”*, after which overnight silanization ensured formation of a hydrophobic monolayer on the surface, also described in “*Single Stiffness Gradient”*. The second gradient treatment, as described in “*Single Wettability Gradient*”, was applied perpendicular to the first gradient, yielding the S and W DOG surface. This second treatment was performed closely before cell culture studies and directly after treatment the freshly treated DOG was immersed in milliQ water to ensure the polar environment inhibited the hydrophobic recovery process.

##### Topography Characterization

The surface T features were characterized using a NanoScope V AFM (Bruker, Billerica, MA, USA) with NanoScope Analysis (also Bruker) as analysis software. A Bruker model DNP‐10 tip of nonconductive silicon nitride was mounted and used to scan (in air mode) and visualize the surface T. A total of 11 positions along the gradient were measured with 2 mm intervals. At every position, 3 measurements were taken at positions directly perpendicular to the gradient direction. Wavelength and amplitude of the topographic wrinkles were analyzed in NanoScope software. Qualitative scanning electron microscopy was performed using a Philips XL30 environmental scanning electron microscope. Five linearly divided areas of 50 μm^2^ along the gradients were scanned at a sampling rate of 0.100 HZ (sampling speed = 6 μm s^−1^).

##### S Characterization

The surface S was assessed by force indentation analysis, measuring the surface Young's modulus, using a Catalyst NanoScope V instrument (Bruker), and a Bruker model RTESPA‐150 tip (tip radius = 8 nm, tip half angle = 12°). The deflection sensitivity of the tips was assessed using a reference glass slide, after which thermal tuning was used to determine the tip cantilever spring constant (k = 5.17 N m^−1^). Quantitative nanomechanical mapping mode was used (Sneddon model) taking force indentation curves over a preset surface area of 500 nm^2^. The complete span of the 2 cm S gradient was divided into five equally spaced positions, measuring at X = 0, 5, 10, 15, and 20 mm, in triplicate rows in the Y direction. The modulus was calculated using the retraction portion of the force curves, assuming a Poisson ratio of 0.5 (incompressible material). Indentation depth ranged from 30 to 70 nm.

##### W Characterization

The surface W was characterized by using an in‐house developed tensiometer, using the sessile drop method with milliQ water. Droplets of ≈5 μL were placed on the samples in the same pattern as described under “surface stiffness”. WCA were obtained using a built‐in MATLAB (MathWorks) program, measuring the interfacial WCA between the surface and the droplet. W measurements were taken directly after plasma treatment.

##### Cell Culture

hBM‐MSCs (Lonza, passage 4) were cultured in growth medium containing alpha‐modified Eagle medium (Gibco), 10% v/v fetal bovine serum (Gibco), 1% P/S (Gibco), and 0.1% v/v ascorbic acid 2‐phosphate (Sigma). The cells were incubated at 37 °C and 5% CO_2_ until they were grown to 80%–90% confluence in T75 culture flasks, before harvesting by trypsin for ≈3 min at 37 °C.

##### Cell‐Screening Studies

As previously reported by us,^[^ ^54^ ^]^ to retain surface hydrophilicity after plasma treatment, all the DOGs possessing a gradient in surface W were stored in MilliQ water directly after plasma treatment, to negate the adverse effect of the nonpolar, air–hydrophilic PDMS interface, the latter of which initiates a hydrophobic recovery process. Afterward, all samples were sterilized by washing with ethanol (70%) three times and washing in phosphate buffered saline (PBS) three times, after which they were stored in sterile 6‐well plates (Greiner CELLSTAR) in PBS until cell seeding. The hBM‐MSCs were seeded at a density of 3 × 10^4^ cells/well, which were cultured for 24 h at 37 °C and 5% CO_2_. Fixation of the cells was done using 3.7% paraformaldehyde (Sigma–Aldrich) solution for 20 min at room temperature. After removal of the fixative, the wells were washed three times in PBS. Next, the cell membrane was permeabilized using 0.5% TritonX‐100 (Sigma–Aldrich) solution for 3 min at room temperature. After this, a solution of 5% bovine serum albumin (BSA) in PBS (PBSA) was added to block nonspecific binding, for 30 min. The PBSA (5%) was removed, after which the primary antibody against vimentin (mouse–antihuman, Sigma‐Aldrich, 1:100 in 1% PBSA) was added and incubated for 1 h in the dark at room temperature. After removal, the secondary fluorescein isothiocyanate‐labeled goat–antimouse antibody (Jackson Immunolab, 1:100) was added, in addition to DAPI and TRITC–phalloidin, staining the nucleus and the cells’ actin filaments, respectively. This last mixture was added to 1% BSA in PBS solution, added to the wells and left to incubate for 1 h in the dark at room temperature. After removal of the staining solution, the wells were washed two times in 1% PBSA for 5 min and one time in PBS for 5 min, after which they were stored in PBS in the dark at 4 °C until microscopic observation.

##### Visualization and Analysis

A Zeiss AxioImnager.Z1 fluorescence inverted microscope was utilized to automatically scan the samples at 10× magnification (air objective), controlled by TissueFAXS software (TissueGnostics GmbH, Vienna, Austria). Image acquisition was done under constant acquisition parameters (exposure time, threshold settings, and gain) and combined using Tissue‐Gnostics software. Cell behavior, concerning cell density, cell area, and vimentin expression were quantified using TissueQuest software. The area of the fluorescent signal was normalized to the number of cells analyzed using the DAPI channel. A selected grid of 7 × 7 regions of analysis (4 mm^2^ per region) was used to analyze the complete 20 × 20 mm^2^ DOG surfaces, taking into account the exponential trend of the gradients.

##### Homogeneous Translation Studies

ROIs 1–6 were found to be reproduced using the following plasma system settings: 1) 90 mTorr pressure for 10 s on flat PDMS; 2) 1000 mTorr pressure for 15 s on a λ = 3.5 μm imprint; 3) 40 mTorr pressure for 15 s on a λ = 3.5 μm imprint; 4) untreated flat PDMS; 5) 1000 mTorr for 10 s on a λ = 10 μm imprint; and 6) untreated T imprint (λ = 10 μm). Plasma treatment conditions to generate either λ = 3.5 μm or λ = 10 μm homogeneous samples were 20% stretch at 25 mTorr for 60 s and 20% stretch at 25 mTorr for 650 s, respectively. Both homogeneous templates were imprinted according to earlier described methodology. Characterization data can be found in Figure S2, Supporting Information. Cell culture studies were performed and visualized in the exact same manner as described earlier.

##### Statistical Analysis

Partial least squares regression was performed on the individual DOGs using the R programming language version 4.3.1 and the “pls” package version 2.8.2. For each DOG, “S‐W”, “T‐W”, “T‐SW”, and “T‐S”, we applied PLSs regression using the three dependent variables and the three independent variables. Each variable was standardized by dividing it by its standard deviation. In cases where one of the predictors was fixed (constant), an offset term was added to avoid numerical errors as the standard deviation is zero. Finally, leave‐one‐out “LOO” cross‐validation was used to potentially determine the number of components to take into account. The plots were created using the R packages “ggplot2” version 3.4.2 and “tikzDevice” version 0.12.4.

Statistical analysis on screening and translation data was conducted with GraphPad Prism 9.5.1.733 software. All data was expressed as mean values (n = 3 and n = 4) ± standard deviation. The “hit” and “translation” data were analyzed using ordinary one‐way analysis of variance (ANOVA), with Bonferroni's multiple comparisons test to determine differences between the groups. The analysis on the translation data was conducted by an ordinary one‐way ANOVA, with Tukey's multiple comparisons test to determine differences between the groups. A *p*‐value of *p* < 0.05 was considered to be statistically significant.

## Conflict of Interest

P.v.R. also is co‐founder, scientific advisor, and share‐holder of BiomACS BV, a biomedical oriented screening company.

## Supporting information

Supplementary Material

## Data Availability

The data that support the findings of this study are available from the corresponding author upon reasonable request.
