# Supplementary material for: Double‐Orthogonal Gradient‐Based High‐Throughput Screening Platform for Studying Cell Response Toward Combined Physicochemical Biomaterial Properties
Source: Small Sci. 2023 Nov 27;4(1):2300172. doi: 10.1002/smsc.202300172 (PMC11935256; doi:10.1002/smsc.202300172)
Supplement: Supplementary file 1 — Supplementary Material [file SMSC-4-2300172-s001.pdf]

SUPPORTING INFORMATION

Double Orthogonal Gradient-based High-throughput screening platform for studying cell response toward combined physicochemical biomaterial properties.

Authors

*Torben A.B. van der Boon, Lisa E. Tromp, Lu Ge, Liangliang Yang, Carlos F. Guimaraes, Philipp T. Kühn, Qihui Zhou, Ruud A. Bank, Theo G. van Kooten, Patrick van Rijn**

T.A.B. van der Boon, L.E. Tromp, dr. P.T. Kühn, prof. R.A. Bank, dr. T.G. van Kooten, dr. P. van Rijn
University of Groningen, University Medical Center Groningen, department of Biomedical Engineering FB-40, W.J. Kolff Institute for Biomedical Engineering and Materials Science, A. Deusinglaan 1, 9713 AV, Groningen, the Netherlands
Email: p.van.rijn@umcg.nl

dr. L. Ge, dr. L. Yang
School of Pharmaceutical Science, Wenzhou Medical University, Wenzhou, Zhejiang 325000, China.

C. F. Guimares
1. 3B's Research Group - Biomaterials, Biodegradables and Biomimetics. Headquarters of the European Institute of Excellence on Tissue Engineering and Regenerative Medicine, University of Minho. AvePark, Parque de Ciência e Tecnologia, Barco, Guimarães 4805-017, Portugal
2. ICVS/3B's - PT Government Associate Laboratory Braga and Guimarães Portugal

dr. Q. Zhou
School of Rehabilitation Sciences and Engineering, University of Health and Rehabilitation Sciences, Qingdao 266071, China.

Supplementary Figures

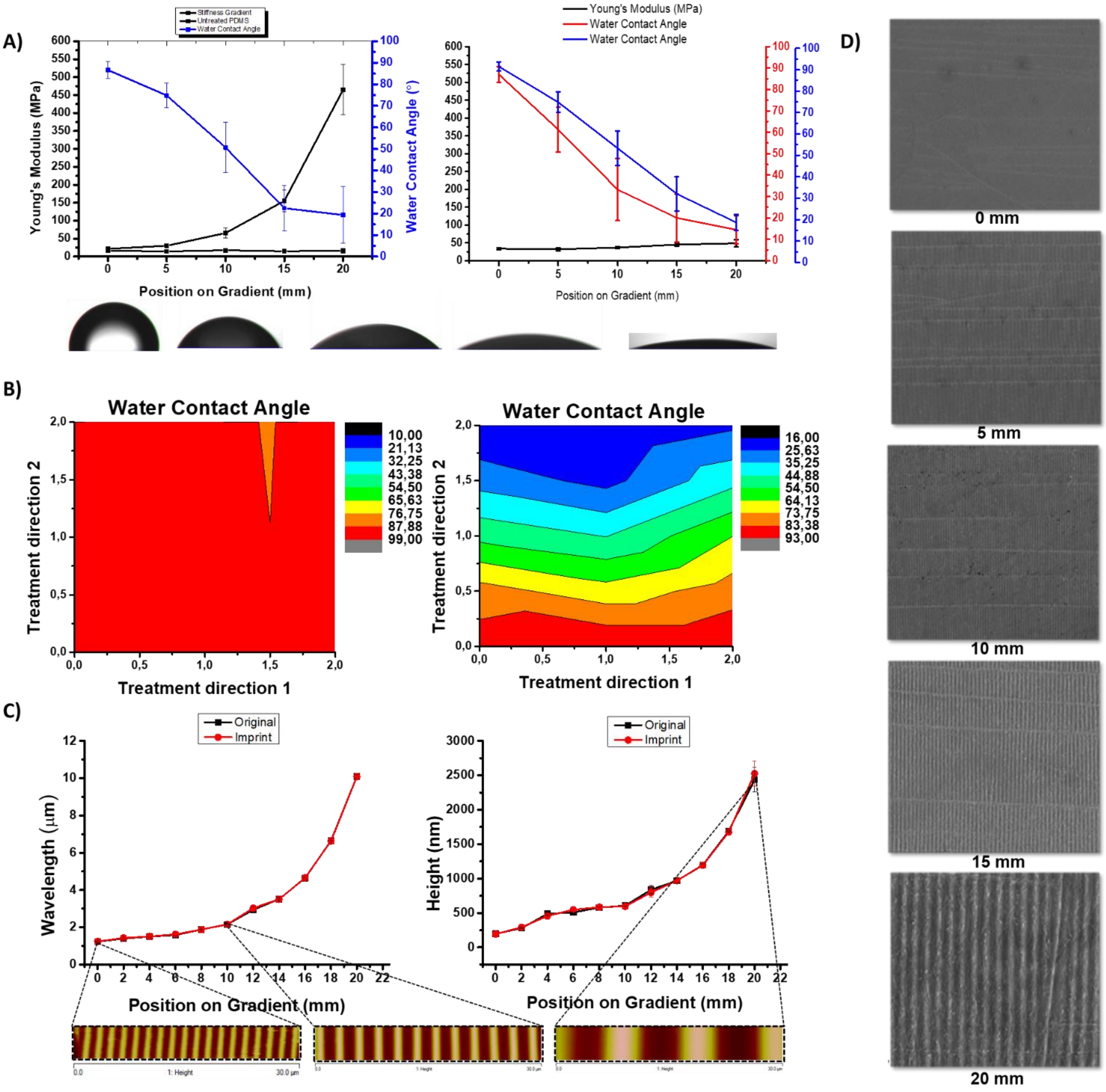

Figure S1. Physicochemical surface gradients of; A) surface stiffness and surface wettability both coupled (left) and decoupled (right) where the blue line represents the wettability gradient on the **S-W** combination and the red line on the **T-W** combination, B) hydrophobic recovery by overnight silanization and linear surface wettability gradient in perpendicular direction of initial treatment, and C) surface topography and imprints thereof in pristine PDMS, with corresponding AFM data D) SEM data (1000 x magnification, spot size: 2, beam current: 3kV).

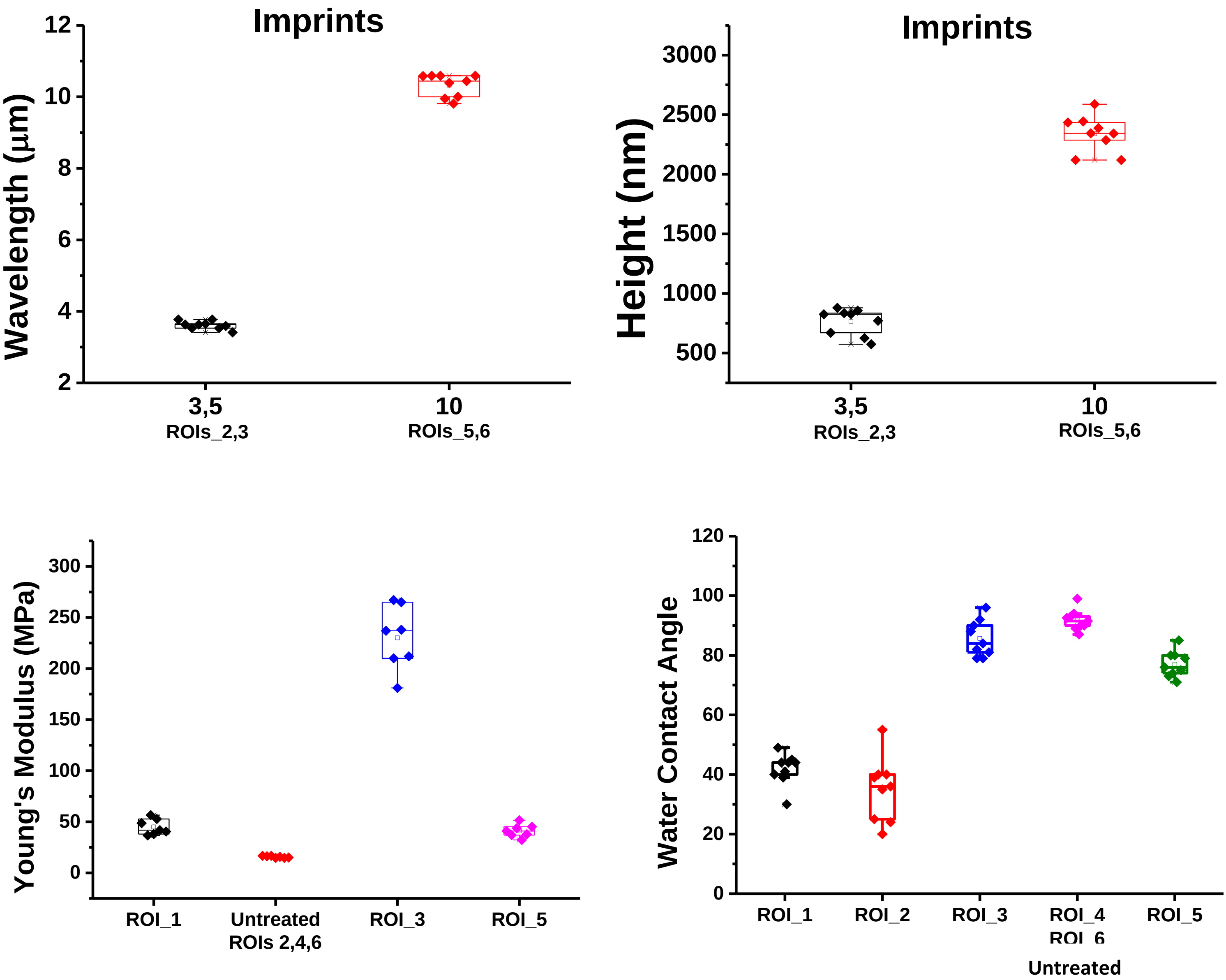

Figure S2. Data on surface physicochemical conditions in translation experiments showing; topography (top), and stiffness and wettability (bottom).

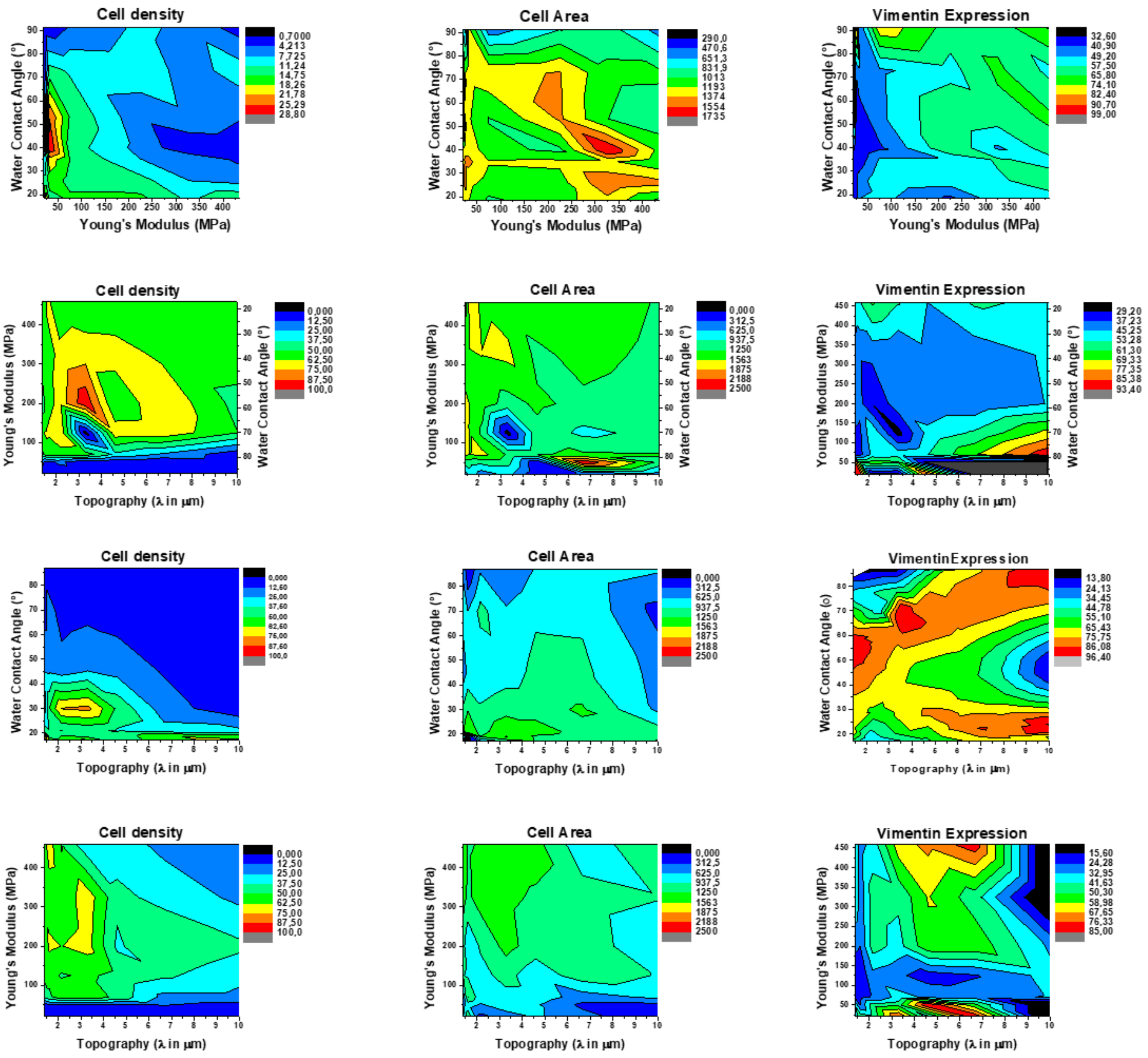

Figure S3. Screening overview of experiment *N1*, showing cell density (cells/mm^2), cell area (μm^2) and vimentin expression (A.U.).

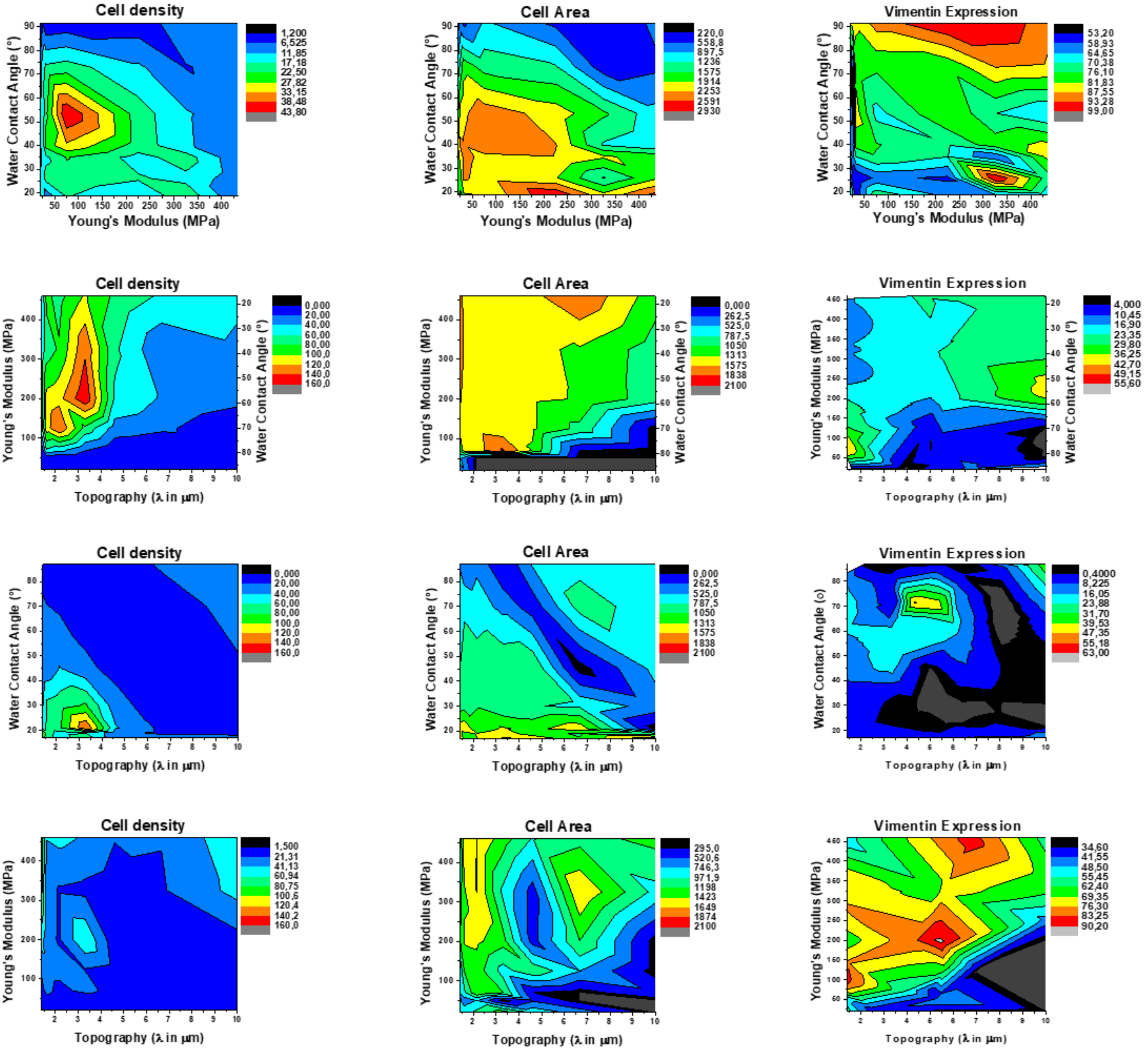

Figure S4. Screening overview of experiment *N2,* showing cell density (cells/mm^2), cell area (µm^2) and vimentin expression (A.U.).

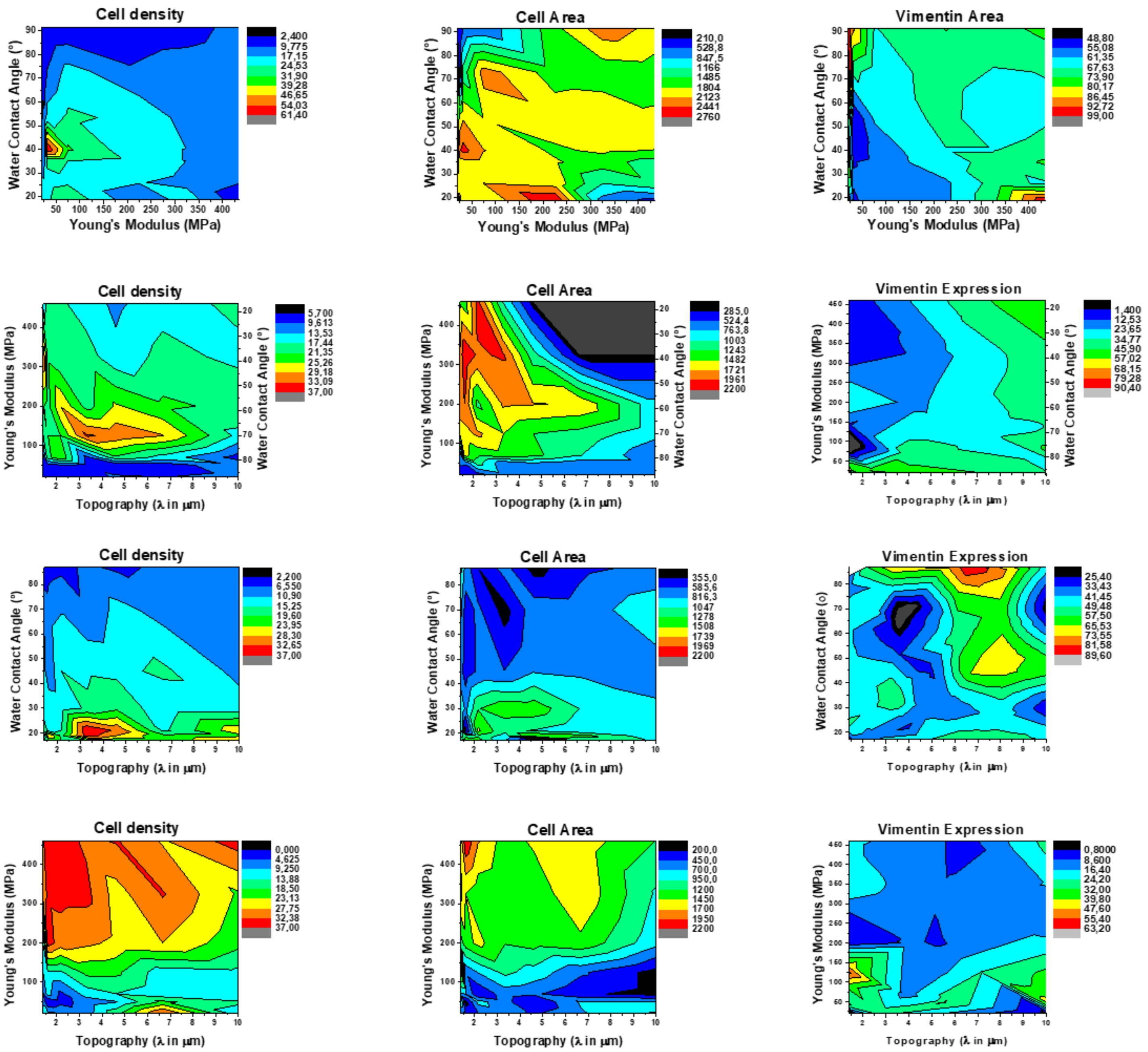

Figure S5. Screening overview of experiment *N3,* showing cell density (cells/mm^2), cell area (μm^2) and vimentin expression (A.U.).

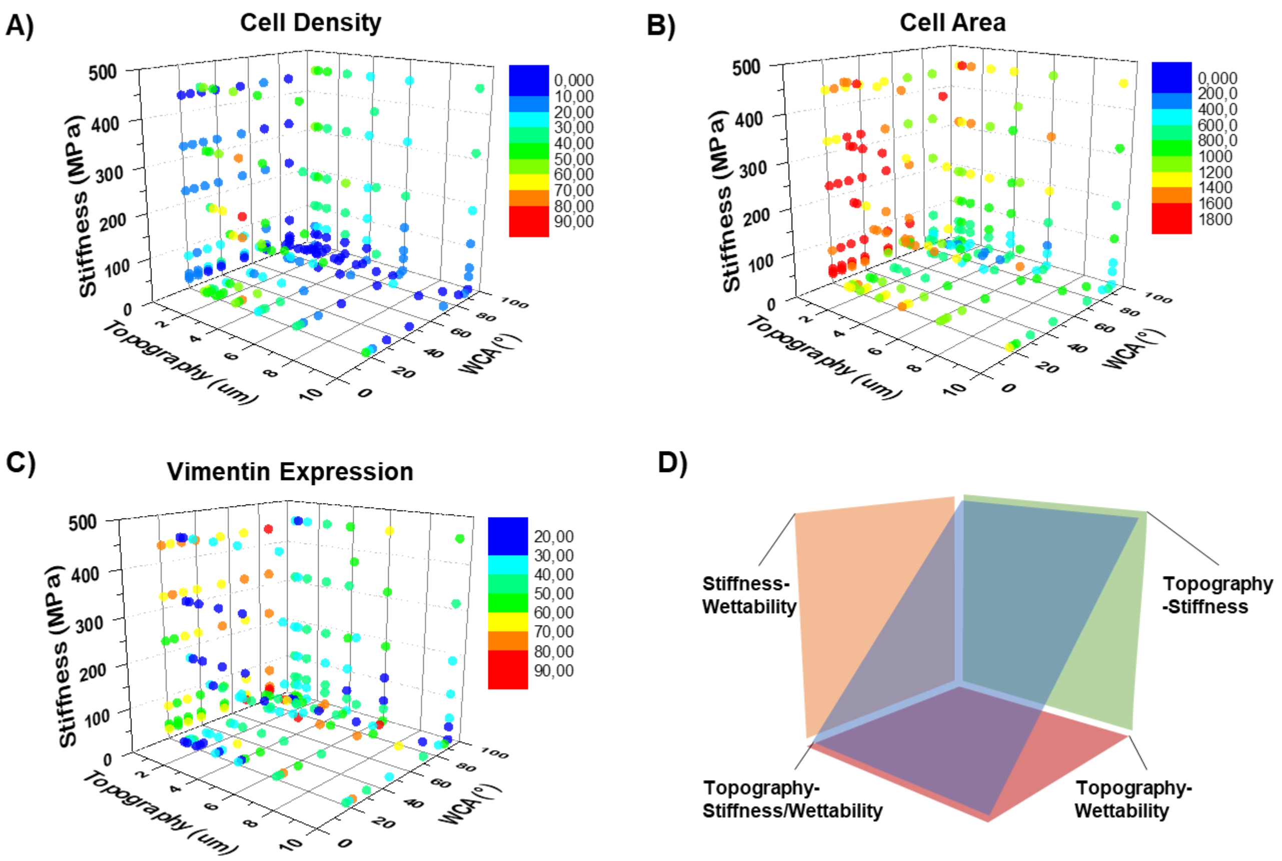

Figure S6. Three-dimensional representations of all averaged data (588, n=3) of the screening output. Shown are cell density (cells/mm^2) (a), cell area in (µm^2) (b), and vimentin expression (A.U.) (c). A visual aid to showcase how the data originating from the platform is organized 3D is shown in (d).

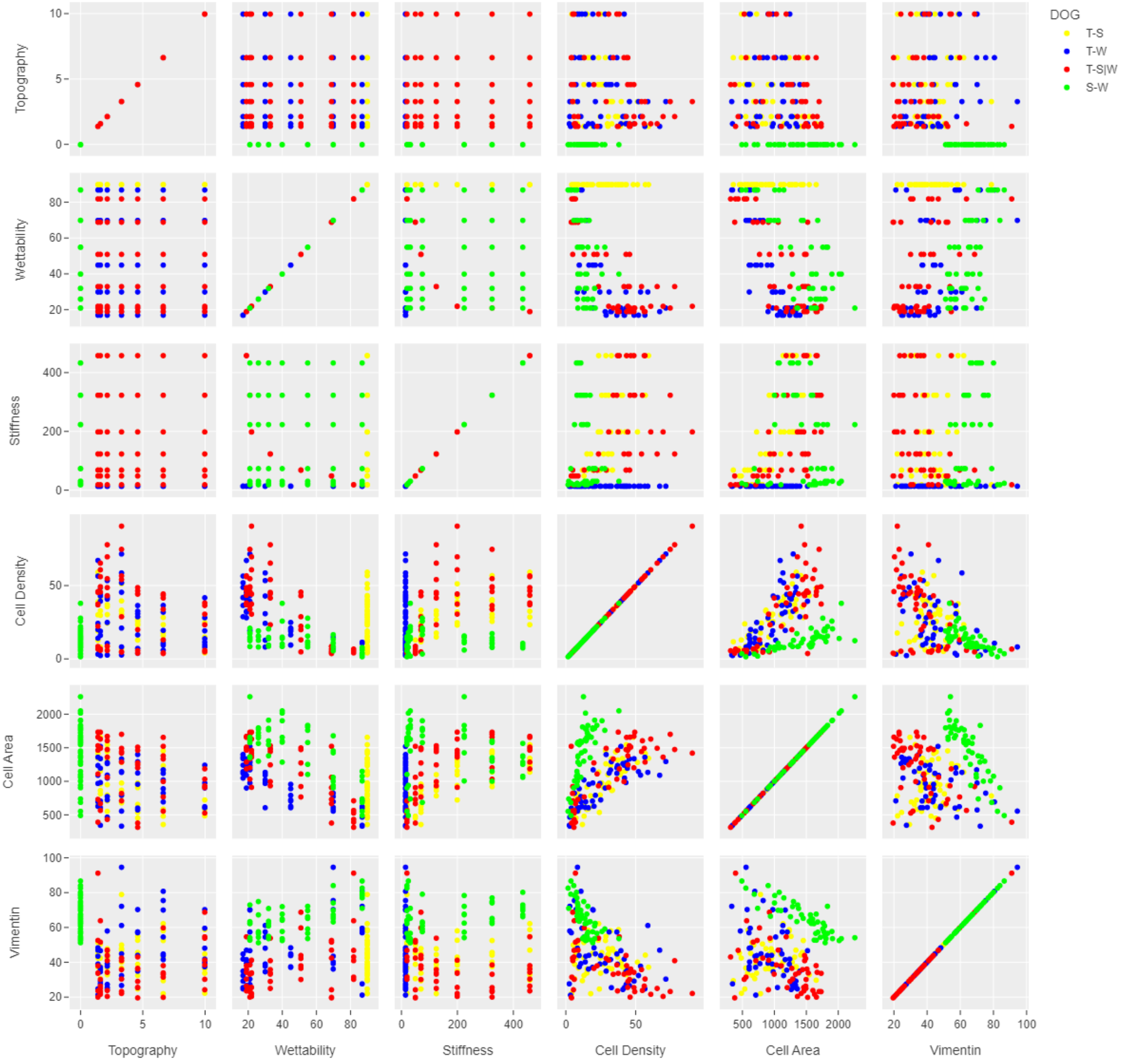

Figure S7. Overview of dataset in scatterplots**.** Depicted are the relationships between surface parameters of topography, wettability, and stiffness, with the assessed cell behavior.

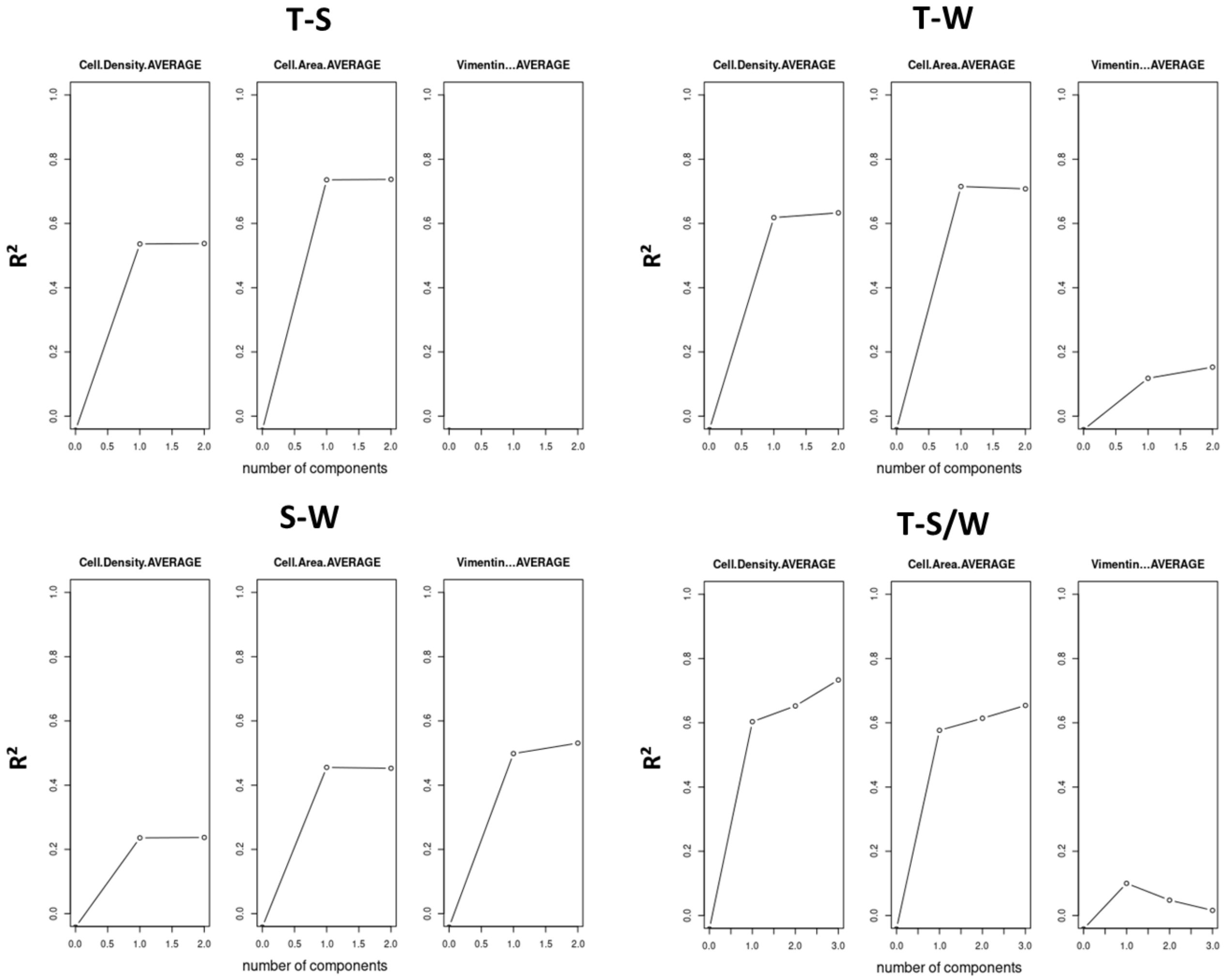

Figure S8. R-squared values and number of components used to describe the date in PLS regression analysis.

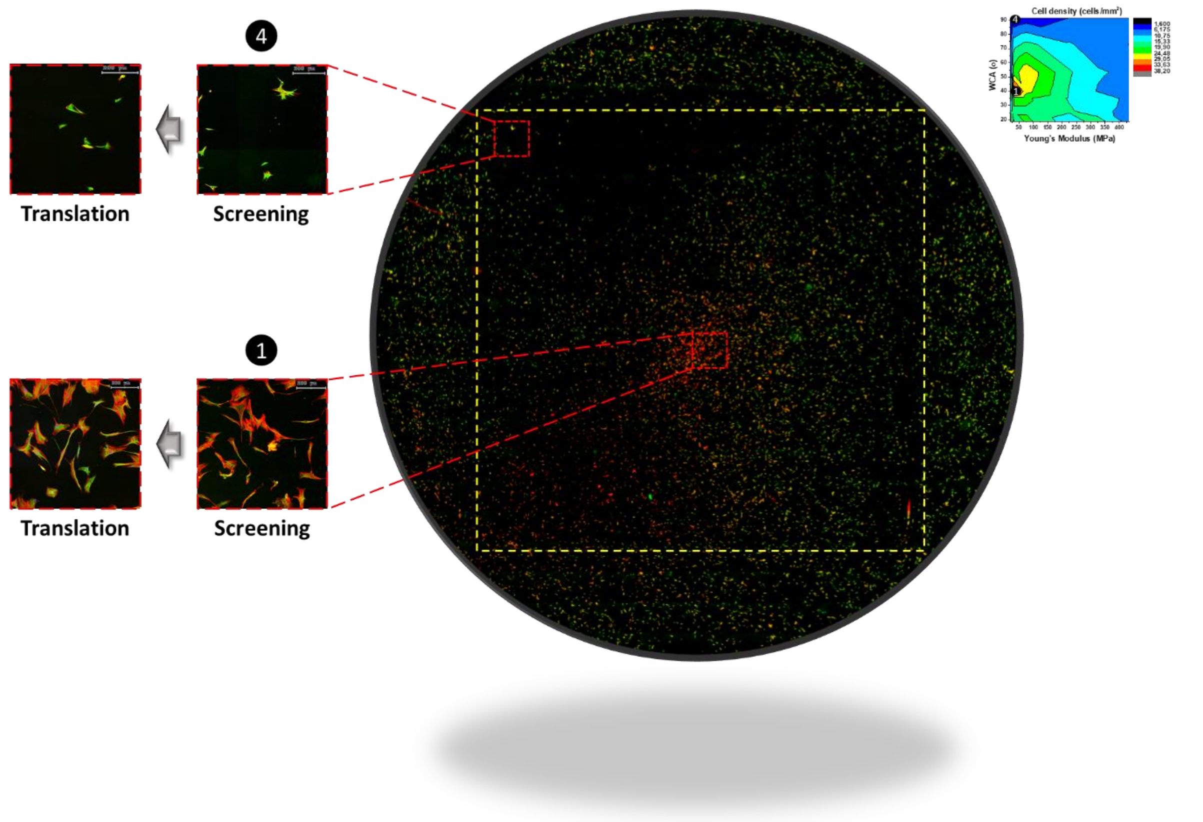

Figure S9. Fluorescent representation of screening and translation overview, showing complete overview of 20 x 20 mm S-W treated surface with adhering MSCs, as well as zoomed in fluorescent images of screening 'hits' 1 and 4 and coupled ROI translation experiments.

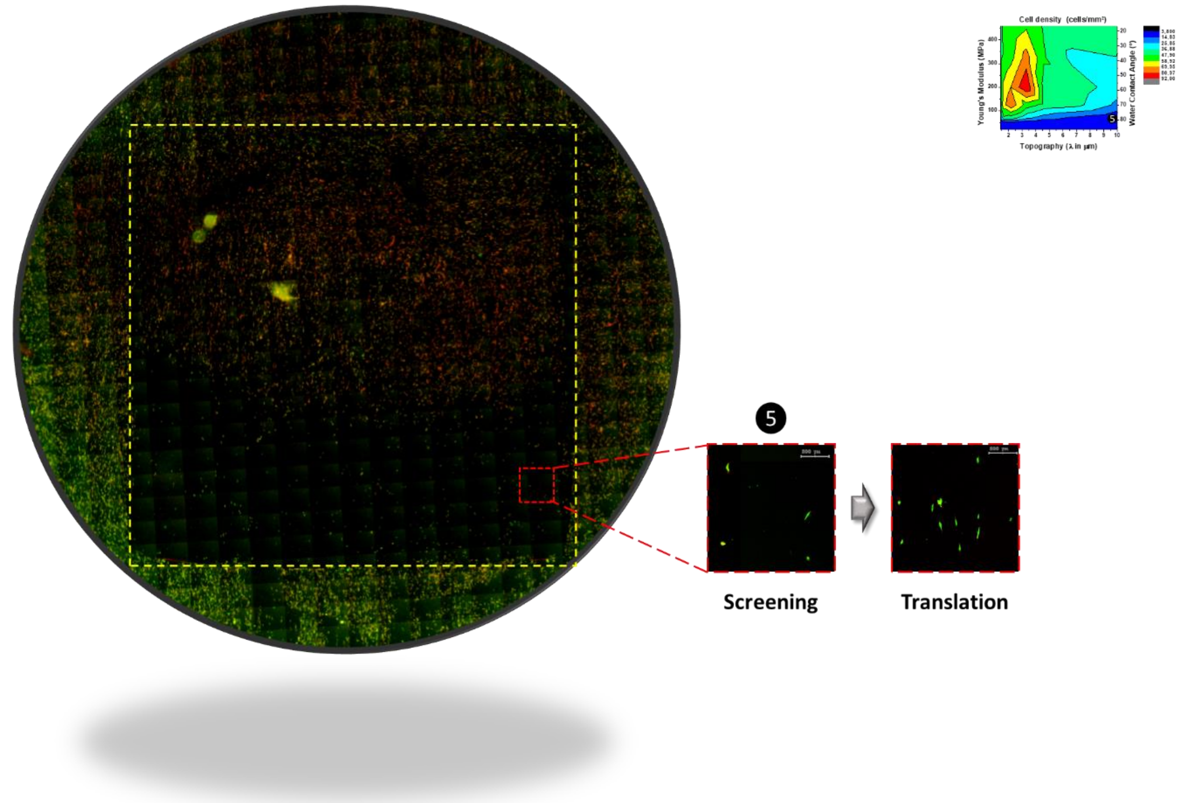

Figure S10. Fluorescent representation of screening and translation overview, showing complete overview of 20 x 20 mm T-S-W treated surface with adhering MSCs, as well as zoomed in fluorescent images of screening 'hit' 5 and coupled ROI translation experiments.

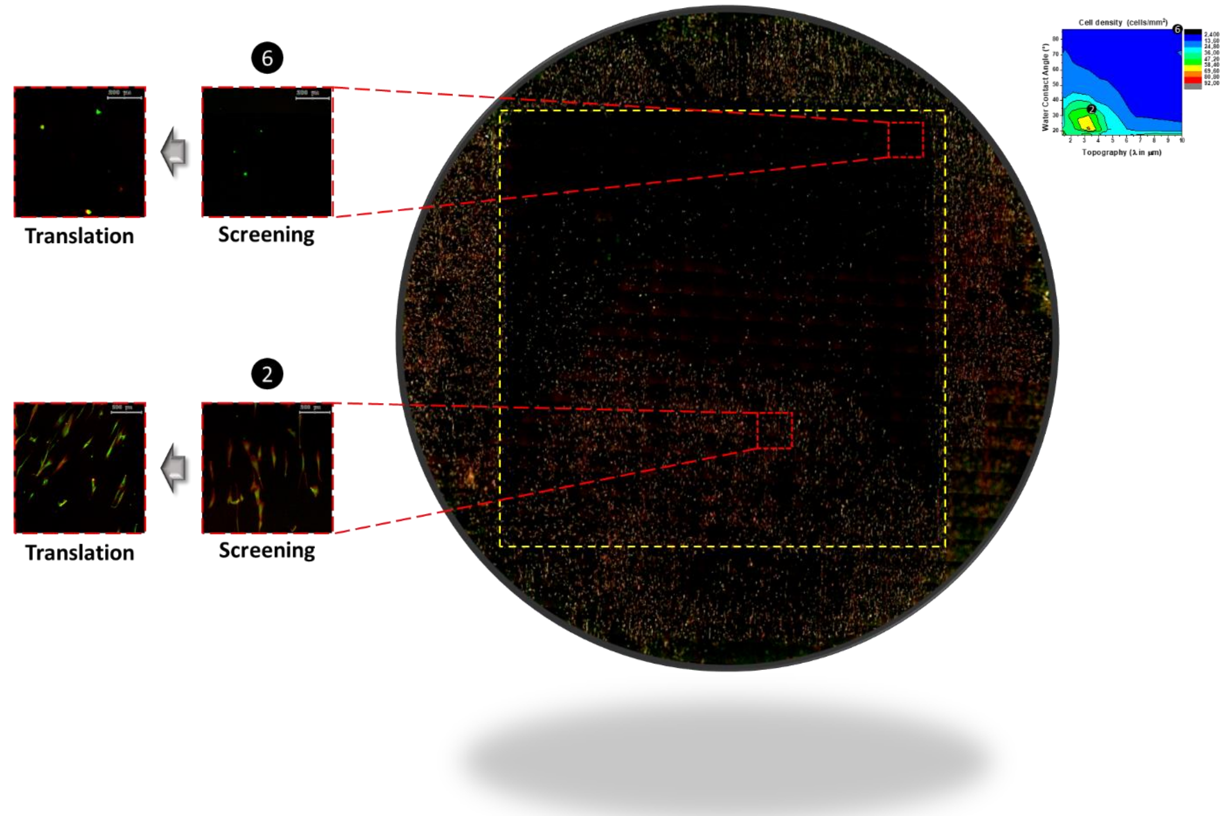

Figure S11. Fluorescent representation of screening and translation overview, showing complete overview of 20 x 20 mm T-W treated surface with adhering MSCs, as well as zoomed in fluorescent images of screening 'hits' 2 and 6 and coupled ROI translation experiments.

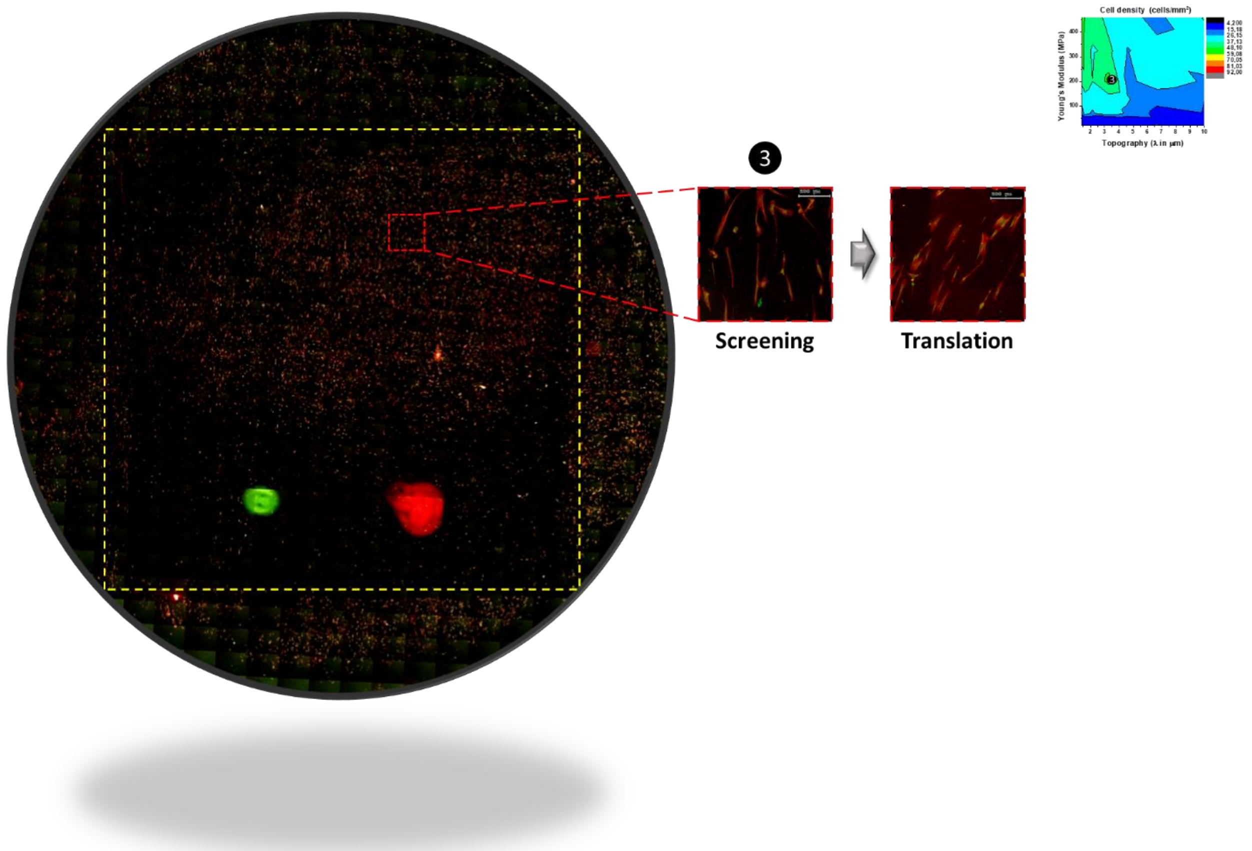

Figure S12. Fluorescent representation of screening and translation overview, showing complete overview of 20 x 20 mm T-S treated surface with adhering MSCs, as well as zoomed in fluorescent images of screening 'hit' 3 and coupled ROI translation experiments.
